# Differences in food consumption between patients with Hashimoto’s thyroiditis and healthy individuals

**DOI:** 10.1038/s41598-020-67719-7

**Published:** 2020-06-30

**Authors:** Dean Kaličanin, Luka Brčić, Katija Ljubetić, Ana Barić, Sanda Gračan, Marko Brekalo, Vesela Torlak Lovrić, Ivana Kolčić, Ozren Polašek, Tatijana Zemunik, Ante Punda, Vesna Boraska Perica

**Affiliations:** 1 Department of Medical Biology, University of Split School of Medicine, Soltanska 2, 21000 Split, Croatia; 2 Department of Clinical Nutrition, Faculty of Health Studies, University of Rijeka, 51000 Rijeka, Croatia; 3 Department of Nuclear Medicine, University Hospital Split, 21000 Split, Croatia; 4 Department of Public Health, University of Split School of Medicine, 21000 Split, Croatia

**Keywords:** Thyroid diseases, Nutrition

## Abstract

Food is considered as important environmental factor that plays a role in development of Hashimoto's thyroiditis (HT). The goal of our study was to identify food groups, assessed by food frequency questionnaire, that differ in consumption frequency between 491 patients with HT and 433 controls. We also analysed association of food groups with the wealth of HT-related clinical traits and symptoms. We found significantly increased consumption of animal fat (OR 1.55, *p* < 0.0001) and processed meat (OR 1.16, *p* = 0.0012) in HT cases, whereas controls consumed significantly more frequently red meat (OR 0.80, *p* < 0.0001), non-alcoholic beverages (OR 0.82, *p* < 0.0001), whole grains (OR 0.82, *p* < 0.0001) and plant oil (OR 0.87, *p* < 0.0001). We also observed association of plant oil consumption with increased triiodothyronine levels in HT patients (β = 0.07, *p* < 0.0001), and, association of olive oil consumption with decreased systolic blood pressure (β = − 0.16, *p* = 0.001) in HT patients on levothyroxine (LT4) therapy. Analysis of food consumption between HT patients with and without LT4 therapy suggest that patients do not tend to modify their diet upon HT diagnosis in our population. Our study may be of relevance to nutritionists, nutritional therapists and clinicians involved in developing dietary recommendations for HT patients.

## Introduction

Environmental factors, in addition to genetic factors, play important role in Hashimoto’s thyroiditis (HT) development^1^. HT is a chronic autoimmune thyroid disorder (AITD) characterized by the production of thyroid autoantibodies against thyroid peroxidase (TPOAb) and thyroglobulin (TgAb)^2^. Other main characteristics of HT include infiltration of lymphocytes and ruination of thyroid tissue, which usually leads to hypothyroidism^3^. This disease predominantly affects female population and is considered as one of the most frequent endocrine disorders^4^. The estimated incidence of HT is 350/100,000 for females and 80/100,000 per year for males^5^. It is most often diagnosed in women between the age of 30 and 60^6^.

High iodine intake (median urinary iodine concentration ≥ 300 μg/L)^7^, especially in areas with sufficient iodine supply, is one of the most known factors that increase the incidence of HT^8^. Also, low vitamin D levels (below 30 ng/mL)^9^ and low selenium intake (bellow 40 µg/day)^10^ are proposed to be risk factors for HT development, albeit a role of these factors in HT still needs to be confirmed. Conversely, alcohol intake was proposed to decrease the risk for HT^11^. Other dietary factors are also considered as potential environmental modifiers of the clinical appearance of HT^12–15^. A diet low in gluten has been recently proposed to have beneficial effects on the disease course in a study of 34 women with HT^13^. Another study of 83 patients with HT indicated that restriction of lactose intake in lactose-intolerant HT patients resulted with decreased thyroid-stimulating hormone (TSH) levels^14^. However, the current knowledge of this topic is scarce and only the following two studies analyzed the association between thyroid function and dietary factors using food frequency questionnaire (FFQ) as an assessment tool to date. A large population-based study of 65,981 participants found an association of the vegan diet with a lower risk of self-reported hypothyroidism^16^. Another recent study showed that higher animal fat and butter intake are associated with the risk of positivity to TPOAb and/or TgAb antibodies using 1,887 participants, whereas diet rich in vegetables, dried fruit, nuts and muesli consumption decreased that risk^17^.

The first goal of our study is to identify specific food groups, assessed by FFQ, that differ in consumption frequency between patients with HT and control subjects. Our second goal is to perform a comprehensive set of analyses to test associations of diet with the wealth of HT-related phenotypes and symptoms. Our third goal is to exploit if HT patients change their diet upon HT diagnosis. The overall aim of our study is to contribute to the knowledge related to diet and HT and may be of relevance to nutritionists, nutritional therapists and clinicians involved in developing dietary recommendations for HT patients.

## Methods

### Study participants

Our study investigated 924 individuals from the region of Split (Southern Croatia), including 491 HT cases and 433 control participants. All procedures have been established according to guidelines of the most relevant thyroid societies (ATA, ETA, Croatian thyroid society). Patients were recruited from the Outpatient clinic for thyroid disorders at the University Hospital Split in the period from 2013 to 2017 and are a part of the Croatian Biobank of patients with Hashimoto’s thyroiditis (CROHT)^18^. The study involved newly diagnosed patients along with patients on follow up. Diagnosis was made by a clinical specialist in nuclear medicine primarily based on clinical examination and thyroid ultrasound. Only individuals with echographic pattern of diffuse thyroid disease (unhomogenous thyroid tissue with diffusely reduced echo levels) were included in the study. Diagnosis of HT was further complemented by evaluation of biochemical measurements. HT was confirmed by at least one of the laboratory parameters falling out of referent ranges: increased TSH and/or decreased thyroid hormones [total triiodothyronine (T3), total thyroxine (T4), free thyroxine (fT4)] and/or increased TPOAb and TgAb autoantibodies. Hormones (TSH, T3, T4, fT4) and levels of thyroid antibodies (TgAb and TPOAb) were measured from the plasma of participants by immunoassay using the fully automated "Liaison" Biomedica Chemiluminescence Analyzer (DiaSorin, Saluggia, Italy). We used the following reference values for hormones and thyroid antibodies levels that are valid for our population: TSH (0.3–3.6 mUI/L), T3 (1.3–3.6 nmol/L), T4 (57.4–161 nmol/L), fT4 (10.1–22.3 pmol/L), TPOAb (1–16 IU/mL), TgAb (5–100 IU/mL). Thyroid ultrasonography was performed using the Medison Accuvix V10 (Samsung Medison Co. Ltd, Seoul 135–280, Korea) high frequency linear probe (8–12 MHz). Thyroid volume was calculated as a sum of volumes of both lobes, where the volume of each lobe was calculated as length × width × depth × 0.479^19^. The body surface area (BSA) in m^2^ was calculated as W^0.425^ × H^0.725^ × 0.007184, where W is the weight (kg) and H the height (cm).

Our HT cases include 289 HT patients without LT4 therapy and 177 HT patients who are taking LT4 therapy (information on LT4 therapy was not available for 25 HT patients). HT patients without LT4 therapy had median TSH levels just above the upper referent values and T3 and T4 levels in normal ranges, indicating that these patients were newly diagnosed or in follow-up care with preserved thyroid function. HT patients with LT4 therapy had median TSH, T3 and T4 levels within the reference ranges, suggesting that their LT4 supplementation was appropriate. Median TgAb/TPOAb levels were increased (above referent values) in both sub-groups of patients. Clinical and sociodemographic characteristics of HT cases are shown in Table 1. A large number of phenotypes was collected from each patient, of which the other relevant ones for this study are: systolic and diastolic blood pressure and the presence/absence of the most common 16 symptoms of hypothyroidism, chosen on the basis of clinical expertise and the data from the literature (weakness, increase in weight, constipation, sensitivity to coldness, cold skin, dry and rough skin, pale skin, loss of skin hair, fragile hair, edema of the eyes, face edema, peripheral edema, harsh voice, slow speech, dyspnea and memory disturbance)^20,21^.

**Table 1:** Clinical and sociodemographic characteristics of 491 HT patients and 433 control participants.

Variable	HT cases	Controls	*p*
	Median (Q1-Q3)	Median (Q1–Q3)	
TSH, mIU/L	3.31 (1.76–5.60)	1.65 (1.13–2.36)	< 0.0001^a^
T3, nmol/L	1.6 (1.30–1.80)	1.7 (1.50 -1.90)	< 0.0001^a^
T4, nmol/L	105 (89.03–118)	115 (98.90–123)	< 0.0001^a^
fT4, pmol/L	12.05 (10.20–13.20)	–	–
TgAb, IU/mL	134 (36.4–420)	7.1 (5–10.15)	< 0.0001 ^a^
TPOAb, IU/mL	212 (27–629)	2.1 (1.20–4.80)	< 0.0001 ^a^
Thyroid volume, cm^3^	10.02 (7.33–13.99)	–	–
Systolic blood pressure, mmHg	120 (110–130)	126 (116–141)	< 0.0001 ^a^
Diastolic blood pressure, mmHg	70 (65–80)	77 (69–83)	< 0.0001 ^a^
BSA, m^2^	1.81 (1.70–1.93)	1.92 (1.76–2.10)	< 0.0001 ^a^
BMI, kg/ m^2^	23.51 (20.81–26.73)	26.26 (23.60–29.37)	< 0.0001 ^a^
Physical activity, score	60 (30–62)	45 (18.75–61)	0.174^a^
Age, years	38 (28–49)	51 (39–60)	< 0.0001 ^a^
**Gender, N (%)**			
Male	36 (7%)	172 (40%)	< 0.0001^b^
Female	455 (93%)	261 (60%)	

*TSH* thyroid-stimulating hormone, *T3* total triiodothyronine, *T4* total thyroxine, *fT4* free thyroxine, *TgAb* thyroglobulin autoantibodies, *TPOAb* thyroid peroxidase autoantibodies, *BSA* body surface area, *BMI* body mass index, *Q1* first quartile, *Q3* third quartile, *p* p value.

^a^Mann–Whitney-U test, ^b^χ^2^-test. We used the following reference values for hormones and thyroid antibodies levels for our population: TSH (0.3–3.6 mUI/L), T3 (1.3–3.6 nmol/L), T4 (57.4–161 nmol/L), fT4 (10.1–22.3 pmol/L), TPOAb (1–16 IU/mL), TgAb (5–100 IU/mL).

Control participants were derived from the population-based “10,001 Dalmatians project” cohort that is a part of the Croatian Biobank program with a large phenotype information on study participants^22^. We selected individuals from the region of Split to match the origin of HT cases. We used all available self-reported phenotype information to exclude individuals from controls that have HT or any other type of thyroid disorder, such as Graves’ disease, thyroid cancer, non-autoimmune hypothyroidism or individuals that used drugs for any type of thyroid disorder. We also used available laboratory measures on thyroid hormones levels (T3, T4, TSH) to keep only individuals whose hormone values fall within the reference range for our population. Finally, we excluded from controls all individuals with positivity to TPOAb (TPOAb level > 16 IU/mL) or TgAb (TgAb level > 100 IU/mL). In this way, we strengthened our control group by minimizing the possibility of having individuals with undiagnosed AITD or any other type of thyroid disorder. Clinical and sociodemographic characteristics of controls are shown in Table 1.

Written informed consent was obtained from all study participants. The study was approved by Ethics Committees from the University of Split, School of Medicine (Classification no. 003-08/14-03/0001; Registry no. 2181-198-03-04-14-0028) and University Hospital Split (Classification no. 530-02/13-01/11; Registry no. 2181-147-01/06/J.B.-14-2). Both Ethics Committees declared that the study is in accordance with the provisions of the Code of Ethics and the Helsinki Declaration.

### Assessment of dietary intake

The FFQ is the most commonly used dietary assessment tool for the evaluation of food consumption and measurement of long-term food intake^23^. Dietary intake of HT patients was assessed using the FFQ that consisted of 51 items concerning foods and beverages. The frequency of intake of each food item was measured using six categories: every day, 2–3 times a week, once a month, once a week, rarely, and never. Participants reported the frequency of consummation of specific food but did not quantify the amount of consumed food (servings). Dietary intake in control participants was assessed using the FFQ that consisted of 54 items regarding foods and beverages. The frequency of food intake was measured using five categories: every day, 2–3 times a week, once a week, rarely, and never. Additionally, there was a question in both FFQs regarding fat consumption with three choices (plant oil, olive oil, and animal fat) and three frequency categories (always, sometimes, and never). Prior analysis, we grouped 48 food items that were common in both questionnaires into 22 food groups (SI Table S1) and we converted the frequency categories into weekly intake for each of the 48 food items (SI Table S2). Our FFQ was not designed to quantify daily intake of nutrients from the food groups.

### Statistical analysis

We performed a case–control analysis to identify specific food groups that differ in consumption frequency between HT cases and controls. Specifically, we assessed the association between weekly intake of 22 food groups and HT by using a logistic regression model, where case/control status was used as the dependent variable and 22 food groups as independent variables, along with age and gender.

We also investigated if two sub-groups of HT patients (depending on LT4 therapy) differ in dietary habits, as this analysis is important for data interpretation. The rationale behind this analysis was to take into consideration possible changes in diet between these two sub-groups (for example, HT patients who were taking LT4 therapy might have changed dietary habits upon disease diagnosis) which could introduce spurious associations for our main case–control analysis. We used a logistic regression model, where therapy status (with/without LT4 therapy) was used as the dependent variable and 22 food groups as independent variables, along with age and gender.

We also tested the association between weekly intake of 22 food groups and HT-related quantitative traits (TSH, T3, T4, fT4, TgAb, TPOAb, thyroid volume, systolic blood pressure, and diastolic blood pressure) using a linear regression model, where a selected quantitative trait was the dependent variable and 22 food groups were independent variables. Normality of distribution of residuals was tested using Kolmogorov–Smirnov test while homogeneity of variance was tested using Levene's test. In each model, we included age, gender, and physical activity (the calculation of physical activity is shown in SI Table S3) as covariates and, depending on a quantitative trait, we included additional covariates: body surface area (BSA) and TSH (for association of food groups with thyroid volume, systolic and diastolic blood pressure), BSA (for the association analysis of food groups with TSH and fT4). Analyses were performed in all HT patients and in the two sub-groups depending on LT4 therapy status. In the group comprising all HT patients, we also included LT4 therapy status as an additional covariate, whereas in the sub-group of HT patients that were on LT4 therapy we included both LT4 therapy dose and the weight ratio as an additional covariate. We considered *p* values corrected for 9 tests (*p* < 0.0056) as statistically significant.

Finally, we assessed the association between 22 food groups and 16 symptoms of hypothyroidism in the sub-group of HT patients without LT4 therapy. For each symptom, we used a logistic regression model where symptom status (with/without symptom) was used as the dependent variable and food groups as independent variables. Age, gender, physical activity, body mass index (BMI) and TSH levels were also included as covariates. The *p* value corrected for 16 symptoms of hypothyroidism (*p* < 0.0031) was considered statistically significant. All statistical analyses were performed using the Statistical Package Software for Social Science, version 16 (SPSS Inc., Chicago, IL, USA).

## Results

**Figure 1:**
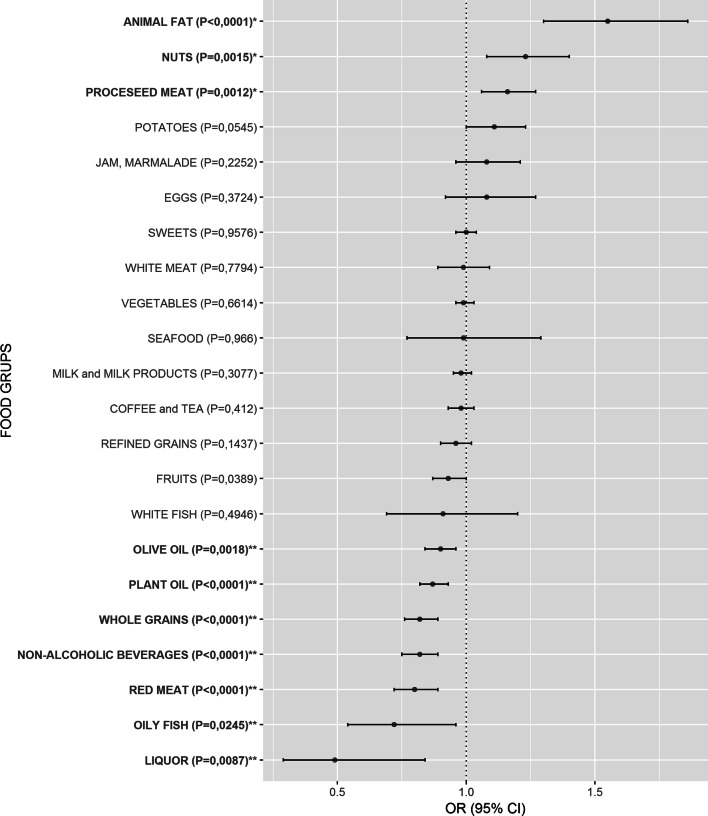
Comparison of weekly intake frequency for 22 food groups between HT patients and controls. Food groups that were more frequently consumed in HT patients than in controls have odds ratios (OR) above 1, whereas food groups with OR below 1 were less frequently consumed in HT patients. If the OR and the lower limit of the 95% confidence interval (CI) were above 1, the food group was significantly more frequently consumed in HT patients than in controls (bolded and marked with *), whereas if the OR and the upper limit of the 95% CI were below 1, the food group was significantly less frequently consumed in HT patients than in controls (bolded and marked with **). Food groups are ordered from the highest to the lowest OR in both subgroups.

The logistic regression analysis revealed that animal fat [OR 1.55 (1.30–1.86), *p* < 0.0001], processed meat [OR 1.16 (1.06–1.27), *p* = 0.0012] and nuts [OR 1.23 (1.08–1.40), *p* = 0.0015] were significantly more frequently consumed in HT patients than in controls, whereas red meat [OR 0.80 (0.72–0.89), *p* < 0.0001], non-alcoholic beverages [OR 0.82 (0.75–0.89), *p* < 0.0001], whole grains [OR 0.82 (0.76–0.89), *p* < 0.0001], plant oil [OR 0.87 (0.82–0.93), *p* < 0.0001], olive oil [OR 0.90 (0.84–0.96), *p* = 0.0018], liquor [OR 0.49 (0.29–0.84), *p* = 0.0087], oily fish [OR 0.72 (0.54–0.96), *p* = 0.0245] and fruits [OR 0.93 (0.87–0.996), *p* = 0.0389] were significantly less frequently consumed in HT patients than in control participants (Fig. 1, SI Table S4). Distribution of intakes in both groups is shown in SI Table S4 as a mean weekly intake for each food group.

**Figure 2:**
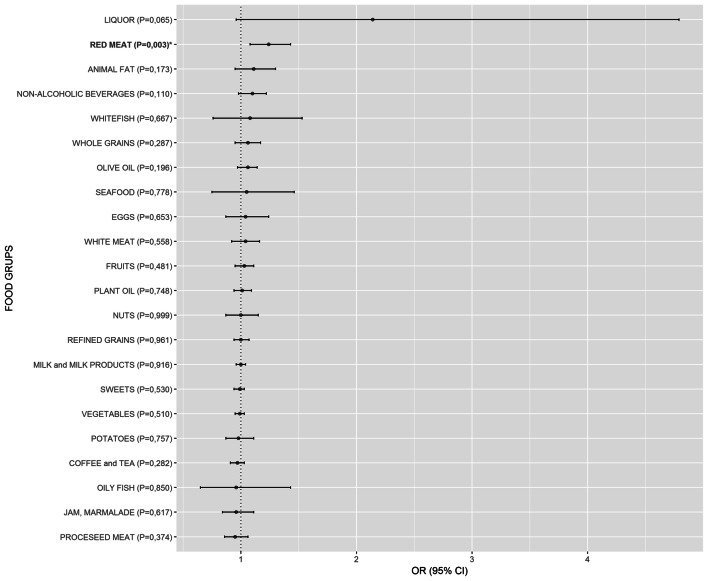
Comparison of weekly intake of 22 food groups between 177 HT patients who were on levothyroxine (LT4) therapy and 289 HT patients without LT4 therapy. Food groups that were more frequently consumed in HT patients on LT4 therapy than in HT patients without LT4 therapy have odds ratios (OR) above 1, whereas food groups with OR below 1 were less frequently consumed in HT patients on LT4 therapy. If the OR and the lower limit of the 95% confidence interval (CI) were above 1, the food group was significantly more frequently consumed in HT patients on LT4 therapy than in HT patients without LT4 therapy (bolded and marked with *). Food groups are ordered from the highest to the lowest OR in both subgroups.

Additionally, our comparison of intake of 22 food groups between HT patients with LT4 therapy and HT patients without LT4 therapy revealed that two sub-groups of HT patients showed no differences in dietary habits except for red meat consumption where patients on LT4 therapy consumed significantly more red meat than HT patients without LT4 therapy [OR 1.24 (1.08–1.43), *p* = 0.003] (Fig. 2, SI Table S5).

Linear regression analysis between weekly intake of 22 food groups and HT-related phenotypes detected a significant positive association between plant oil consumption and T3 levels in the group including all HT patients (β = 0.07, *p* < 0.0001) and in the sub-group of HT patients that were on LT4 therapy (β = 0.10, *p* = 0.001). We also found a negative association between the consumption of olive oil and systolic blood pressure in the sub-group of HT patients that were taking LT4 therapy (β = − 0.16, *p* = 0.001). All other close-to-significant observations are reported in SI Table S6.

The logistic regression between 22 food groups and 16 symptoms of hypothyroidism showed that food groups, in general, are not associated with symptoms in HT patients without therapy. The only exception was our observation of a significant positive association between the consumption of fruit and constipation [OR 1.38 (1.12–1.70), *p* = 0.002]. All other close-to-significant observations are listed in SI Table S7.

## Discussion

As dietary habits may play a role in HT disease development^13–15^, we performed a set of analyses to detect food groups that: (a) differ between HT patients and controls and (b) may be associated with clinical aspects of HT. We found animal fat, processed meat and nuts, as more frequently consumed food groups in HT patients than in controls. Conversely, red meat, non-alcoholic beverages, whole grains, plant oil, olive oil, liquor, oily fish, and fruits were less frequently consumed in HT patients than in controls. Furthermore, this study detected a positive association between plant oil consumption and T3 levels in HT patients and a negative association between olive oil consumption and systolic blood pressure in the sub-group of HT patients that were on LT4 therapy. Another interesting observation of this study is the lack of association between the 16 symptoms of hypothyroidism with food groups, with the exception of the positive association between fruit consumption and constipation in the group of HT patients without LT4 therapy. Finally, we do not observe that patients with LT4 therapy are prone to change their diet due to disease presence. In the text below, we will discuss observed significant associations according to food group.

### Food groups that are more frequently consumed in HT patients

#### Animal fat

The most significant result of our study was observation of significantly increased consumption of animal fat in HT patients in comparison to controls (Fig. 1, SI Table S4). It is important to have in mind that by animal fat we consider fat that is used in cooking/preparing meals excluding all other fat that can be consumed through red meat or other meat products. This result is in line with and could be considered as replication of the result of the recent population-based study of 1887 participants from south Croatia that found association of animal fat consumption with increased TPOAb and/or TgAb positivity^17^, key characteristics of HT^2^. Triglycerides in animal fats, mainly contain saturated (SFA) and monounsaturated fat acids (MUFA)^24–26^. It is known that SFAs may induce development and affect progression of many chronic diseases through inflammatory response^27^. Two recent studies showed that high-fat diet causes thyroid dysfunction in rats and also induces hypothyroidism by decreasing total T4 and fT4 with increasing TSH levels^28,29^. These studies suggested that excess intake of animal fat contributes to pathogenesis of hypothyroidism. Our results are in line with these studies, although this result should not be taken as a suggestion to restrict animal fat consumption until further interventional studies are performed.

#### Processed meat

Processed meat in our study (bacon, sausages and salami) consists mostly of fat, then proteins and in very small amount carbohydrates^25,26^. Other ingredients of processed meat include nitrates and nitrites, which are used as additives for curing meat products, together with sodium^30^. A recent systematic review on animal studies showed that high exposure to dietary nitrate or nitrite induces histomorphological changes in thyroid tissue and a decrease in serum levels of thyroid hormones^31^. It is suggested that high exposure to nitrate/nitrite could inhibit iodine uptake by binding to the sodium-iodide symporter on the surface of thyroid follicles^32,33^.

#### Nuts

We believe that increased consummation of nuts in HT cases may be false positive finding. We base this statement on the recently observed trend of increased nut consumption in the city of Split^34,35^, from which both of our cohorts (HT patients and controls) were derived. More specifically, a comparison of daily nuts consumption between two population-based cohorts from Split, one recruited in 2008–2009 and the other in 2012–2013, showed an increase in nuts consumption from 7 to 11%, respectively^34,35^. Our control group was formed from the former population-based study whereas our HT case cohort was formed during 2015–2016. Therefore, an observed increase of 2.3% in nuts consumption between HT cases and controls in our study may purely reflect the generally established trend of increase in nut consumption in our population. It is important to state that our FFQ did not distinguish between different types of nuts (raw, salted, roasted). Due to all abovementioned reasons, the interpretation of results regarding nuts is difficult and should be taken with caution.

### Food groups that are less frequently consumed in HT patients

#### Red meat

Red meat is a good source of selenium^36^, iron, and zinc^37^, which are essential for the normal function of thyroid hormone synthesis^38^. Additionally, red meat is rich in vitamin B12^37^ whose deficiency is associated with AITD^39^. Unlike processed meat, fresh red meat contains less fat^25,26^ and does not have additives (nitrites, nitrates and sodium). However, for the purposes of our analyses, red meat included several types of meat (pork, veal, beef, lamb and internal organs) that may contain various amounts of animal fat, which is a limitation factor for interpretation of results. An important remark, we have compared intake of food groups between two sub-groups of HT patients (those with and without LT4 therapy) and found unequal red meat consumption (Fig. 2, SI Table S5), however, both groups of patients still consume less red meat than controls, thus excluding a potential spurious result.

#### Non-alcoholic beverages

We also found that HT patients consumed significantly less frequently non-alcoholic beverages. Of these, participants most frequently consumed a domestic fruity flavored multivitamin instant drink called Cedevita that contains 9 vitamins (vitamin C, E and B complex: B1, B2, B3, B5, B6, B9 and B12) in 50% of recommended daily allowances (RDA). There is literature record of vitamin deficiencies (vitamin C, A, D and B complex) in patients with thyroid diseases. An anti-inflammatory diet, rich in vitamins and minerals, is recommended as a diet therapy for HT^40,41^. Additionally, recent study showed that vitamin C intake in AITD patients results in significant lowering of TPOAb antibodies^42^.

#### Whole grains

Whole grains are rich in dietary fibers, source of energy for cecum and colon-residing microbiota that metabolize complex carbohydrates and also produce short-chain fatty acids (SCFAs), which are all important for metabolism, cell proliferation and immune system^43^. Consumption of whole grains, and gluten-rich refined grains, was significantly higher in controls than in HT cases. This is an important observation because it does not support the proposition that a gluten free diet is beneficial to HT patients, as is usually recommended^44,45^. In line with these results, a recent study showed that muesli consumption, which consists mainly of whole grains^46^ was associated with a decreased risk of positivity to TPOAb or TgAb antibodies^17^. Further large randomized studies are needed to give a conclusive answer about effects of gluten reduction in HT patients.

#### Plant oil

Plant oil, especially cold pressed oil, such as sunflower oil, pumpkin seed oil, and olive oil are rich in bioactive components (polyunsaturated fatty acids (PUFA), tocopherols and various phenols) that are associated with anti-inflammatory properties and reduction of oxidative stress^47^. Sunflower oil, the most frequently used type of refined plant oil in Croatia, is a natural source of powerful antioxidant vitamin E^48^.

#### Olive oil

Olive oil, rich in phenols and oleic acid, was suggested as a dietary factor that may induce clinical benefits in some autoimmune diseases, such as systemic lupus erythematosus and rheumatoid arthritis^49,50^. Oleocanthal is a natural anti-inflammatory compound from extra virgin cold pressed olive oil that has ibuprofen-like activity^51^. In fact, many studies have reported anti-inflammatory and immunomodulatory effects from the regular consumption of olive oil^52–54^.

#### Liquor

Moderate alcohol consumption was already proposed to decrease the risk for HT, which is also in line with our results^55^.

#### Oily fish

Oily fish consumption or supplementation with omega-3 PUFAs, eicosapentaenoic acid (EPA) and docosahexaenoic acid (DHA) may reduce inflammation and are of clinical relevance for various autoimmune disorders^56–59^. Importantly, a recent study reported an association between omega-3-rich oily fish intake and decreased serum thyroid autoantibodies levels during pregnancy and suggested dietary prophylaxis against thyroid-related postpartum problems^60^. Oily fish and other seafood are also good source of selenium, iodine, iron and zinc^25,61^ which are known that could play a beneficial role on a AITD^62,63^.

#### Fruits

Finally, beside already mentioned beneficial roles of vitamins, minerals and dietary fibers, fruits additionally contain phytochemicals such as polyphenolic compounds that are known for their anti-inflammatory and antioxidant effects on human health^64^.

### Dietary recommendations

In this paragraph, we briefly discuss our main findings with respect to dietary recommendations. A limitation is that we can discuss our results in the light of the frequency of food intake and not the quantity of intake. According to National dietary guidelines for Croatia^65^, as our main resource, and United States^66^ (Mediterranean-Style Eating Pattern), a lower intake of animal fat and processed meat is identified as healthy eating patterns. Although animal fat is rarely consumed in HT patients (mean weekly intake value 0.9, SI Table S4) it is still consumed over 50% more frequently (OR 1.55) than in control participants (mean value 0.5). Processed meat is also not commonly consumed food, in both studied groups, but it is still consumed more often in HT cases. Recommendations for daily consummation of nuts are not reached in both groups. Red meat consummation is within the recommended intake of 3–4 servings per week in both groups. Consummation of liquor is within recommendations in both groups. Lower consummation of non-alcoholic beverages is recommended, and while we do observe uncommon intake of non-alcoholic beverages in both groups, consummation is higher in controls. Importantly, food groups that are recommended for consummation on daily basis, such as whole grains, plant oil, olive oil and fruits, are consumed more often in controls, albeit without reaching recommendations. Finally, recommendations for oily fish consummation of at least once per week are reached in controls but not in HT cases.

### Association between food group consumption and clinical aspects of HT

We observed a significant positive association between plant oil consumption and T3 levels in all HT patients and in the sub-group of HT patients that were on LT4 therapy. Our observation may be of particular relevance to HT patients, especially those with more pronounced thyroid dysfunction and requires further confirmation. This observation is also in line with the results of our case–control analyses, as discussed above.

We also observed a significant negative association between olive oil consumption and systolic blood pressure in the sub-group of HT patients that were on LT4 therapy. It was found that hypothyroid individuals have elevated 24-h systolic blood pressure in comparison with individuals without diagnosed hypothyroidism^67^. A consumption of olive oil was previously shown to be associated with a reduction of systolic blood pressure in hypertensive and healthy individuals^68,69^. In sum, our study observes that olive oil intake may be associated with positive health outcomes by two lines of evidence: increased consumption in controls and a negative association with systolic blood pressure in HT patients.

Another important result of this study is the lack of association between food groups and 16 symptoms of hypothyroidism^20,21^ that were assessed in the group of HT patients without LT4 therapy indicating that food consumption does not affect reported symptoms in HT patients. The exception was an observation of a positive association between fruit consumption and constipation. Although fruit consumption is regularly associated with decreased constipation^70,71^, we found the contradictory result. A possible explanation is that patients with constipation increased their fruit intake to prevent constipation symptoms. The same explanation has already been found and reported in one study^72^.

This study has several weaknesses that should be mentioned. This is a cross-sectional, observational study that is not designed to give a causal connection between the consumption of food groups and HT development^73^. Future interventional trials may provide the final conclusion on the causality of the observed associations of food groups and HT. For the same reason, our results should not be taken as a straightforward suggestion for changing dietary habits in HT patients. Another limitation includes the difficulty to accurately measure food-intake consumption in large cohorts. In addition, our FFQ was not designed to collect quantitative data on dietary intake, therefore we were not able to perform quantitative daily assessment of food intake and to calculate nutrient composition. An additional limitation of this study is relevant to the analysis of the association between dietary patterns and 16 symptoms of hypothyroidism. Our questionnaire was designed to collect information on the presence/absence of 16 symptoms of hypothyroidism^20,21^, but not to collect information on symptom severity. The information on symptom severity would potentially refine the analysis of the effects of food on symptoms of hypothyroidism. On the other hand, the advantages of our study are that we used a large study group of stringently diagnosed HT patients with measures for many clinically-relevant phenotypes. Also, we carefully selected our control group to avoid analysis of control participants with any thyroid dysfunction. To our knowledge, this is the first study that has investigated differences in food consumption between HT patients and control participants.

In conclusion, we have performed the first study that exploits differences in consumption of food groups between large cohorts of HT patients and controls. Our main findings include observation of increased consumption of animal fat and processed meat in HT patients, but also an increased consumption of several food groups (red meat, non-alcoholic beverages, whole grains, plant oil, olive oil, liquor, oily fish and fruits) in controls. Our study also showed that HT patients are not prone to change their diet upon disease diagnosis, as we have found that two sub-groups of HT patients (depending on LT4 therapy) have no main differences in dietary habits, except for red meat consumption. None of the scientific societies have made dietary recommendations for patients with HT, yet these are highly awaited and still need to be developed. Our study contributes to this aim and increases the, currently limited, knowledge related to diet and HT. Our findings may be of particular relevance to nutritional therapists and clinicians involved in treatment of patients with HT.

## Supplementary information

Supplementary information

## Data Availability

All data is available from the corresponding author upon reasonable request.

## References

[1] Brix TH, Hegedüs L (2012). Twin studies as a model for exploring the aetiology of autoimmune thyroid disease. Clin. Endocrinol..

[2] Zaletel K, Gaberscek S (2011). Hashimoto's thyroiditis: From genes to the disease. Curr. Genom..

[3] Sweeney LB, Stewart C, Gaitonde DY (2014). Thyroiditis: An integrated approach. Am. Fam. Physician.

[4] McLeod DS, Cooper DS (2012). The incidence and prevalence of thyroid autoimmunity. Endocrine.

[5] McGrogan A, Seaman HE, Wright JW, de Vries CS (2008). The incidence of autoimmune thyroid disease: A systematic review of the literature. Clin. Endocrinol..

[6] Mincer DL, Jialal I (2020). StatPearls.

[7] Zimmermann MB, Boelaert K (2015). Iodine deficiency and thyroid disorders. Lancet Diabetes Endocrinol..

[8] Zaletel K, Gaberscek S, Pirnat E (2011). Ten-year follow-up of thyroid epidemiology in Slovenia after increase in salt iodization. Croat. Med. J..

[9] Tamer G, Arik S, Tamer I, Coksert D (2011). Relative vitamin D insufficiency in Hashimoto's thyroiditis. Thyroid.

[10] Ventura M, Melo M, Carrilho F (2017). Selenium and thyroid disease: From pathophysiology to treatment. Int. J. Endocrinol..

[11] Effraimidis G, Wiersinga WM (2014). Mechanisms in endocrinology: Autoimmune thyroid disease: Old and new players. Eur. J. Endocrinol..

[12] Bhatia SK, Rose NR, Schofield B, Lafond-Walker A, Kuppers RC (1996). Influence of diet on the induction of experimental autoimmune thyroid disease. Proc. Soc. Exp. Biol. Med..

[13] Krysiak R, Szkrobka W, Okopien B (2018). The effect of gluten-free diet on thyroid autoimmunity in drug-naive women with Hashimoto's thyroiditis: A pilot study. Exp. Clin. Endocrinol. Diabetes..

[14] Asik M (2014). Decrease in TSH levels after lactose restriction in Hashimoto's thyroiditis patients with lactose intolerance. Endocrine.

[15] Wichman J, Winther KH, Bonnema SJ, Hegedus L (2016). Selenium supplementation significantly reduces thyroid autoantibody levels in patients with chronic autoimmune thyroiditis: A systematic review and meta-analysis. Thyroid.

[16] Tonstad S, Nathan E, Oda K, Fraser G (2013). Vegan diets and hypothyroidism. Nutrients.

[17] Matana A (2017). Dietary factors associated with plasma thyroid peroxidase and thyroglobulin antibodies. Nutrients.

[18] Brcic L (2019). Genome-wide association analysis suggests novel loci for Hashimoto's thyroiditis. J. Endocrinol. Invest..

[19] Brunn J (1981). Volumetric analysis of thyroid lobes by real-time ultrasound (author's transl). Dtsch. Med. Wochenschr..

[20] Chaker L, Bianco AC, Jonklaas J, Peeters RP (2017). Hypothyroidism. Lancet.

[21] Barić A (2019). Thyroglobulin antibodies are associated with symptom burden in patients with Hashimoto's thyroiditis: A cross-sectional study. Immunol. Invest..

[22] Rudan I (2009). "10001 Dalmatians:" Croatia launches its national biobank. Croat. Med. J..

[23] Garcia-Larsen V (2011). Use of a common food frequency questionnaire (FFQ) to assess dietary patterns and their relation to allergy and asthma in Europe: Pilot study of the GA2LEN FFQ. Eur. J. Clin. Nutr..

[24] Lísa M (2011). Characterization of fatty acid and triacylglycerol composition in animal fats using silver-ion and non-aqueous reversed-phase high-performance liquid chromatography/mass spectrometry and gas chromatography/flame ionization detection. J. Chromatogr. A.

[25] Perloff BP, Rizek RL, Haytowitz DB, Reid PR (1990). Dietary intake methodology. II. USDA's Nutrient Data Base for Nationwide Dietary Intake Surveys. J. Nutri..

[26] Kaić-Rak A, Antonić K (1990). Tablice o sastavu namirnica i pića.

[27] Rocha DM, Caldas AP, Oliveira LL, Bressan J, Hermsdorff HH (2016). Saturated fatty acids trigger TLR4-mediated inflammatory response. Atherosclerosis.

[28] Shao SS (2014). Dietary high-fat lard intake induces thyroid dysfunction and abnormal morphology in rats. Acta Pharmacol. Sin..

[29] Zhang X (2018). A high-fat diet rich in saturated and mono-unsaturated fatty acids induces disturbance of thyroid lipid profile and hypothyroxinemia in male rats. Mol. Nutr. Food Res..

[30] Honikel KO (2008). The use and control of nitrate and nitrite for the processing of meat products. Meat Sci..

[31] Bahadoran Z (2015). Is dietary nitrate/nitrite exposure a risk factor for development of thyroid abnormality? A systematic review and meta-analysis. Nitric Oxide Biol. Chem..

[32] Lee C, Weiss R, Horvath DJ (1970). Effects of nitrogen fertilization on the thyroid function of rats fed 40 percent orchard grass diets. J. Nutr..

[33] Bloomfield RA, Welsch CW, Garner GB, Muhrer ME (1961). Effect of dietary nitrate on thyroid function. Science (New York, N.Y.).

[34] Kolcic I (2016). Mediterranean diet in the southern Croatia—does it still exist?. Croat. Med. J..

[35] Relja A (2017). Nut consumption and cardiovascular risk factors: A cross-sectional study in a mediterranean population. Nutrients.

[36] Ferguson LR (2010). Meat and cancer. Meat. Sci..

[37] Mann N (2000). Dietary lean red meat and human evolution. Eur. J. Nutr..

[38] Zimmermann MB, Kohrle J (2002). The impact of iron and selenium deficiencies on iodine and thyroid metabolism: Biochemistry and relevance to public health. Thyroid.

[39] Ness-Abramof R (2006). Prevalence and evaluation of B12 deficiency in patients with autoimmune thyroid disease. Am. J. Med. Sci..

[40] Ihnatowicz P, Drywień M, Wątor P, Wojsiat J (2019). The importance of nutritional factors and dietary management of Hashimoto’s thyroiditis. Ann. Agric. Environ. Med..

[41] Kawicka A, Regulska-Ilow B (2015). Metabolic disorders and nutritional status in autoimmune thyroid diseases. Postepy Hig. Med. Dosw. (Online).

[42] Karimi F, Omrani GR (2018). Effects of selenium and vitamin C on the serum level of antithyroid peroxidase antibody in patients with autoimmune thyroiditis. J. Endocrinol. Invest..

[43] Sang S, Landberg R (2017). The chemistry behind health effects of whole grains. Mol. Nutr. Food Res..

[44] Akcay MN, Akcay G (2003). The presence of the antigliadin antibodies in autoimmune thyroid diseases. Hepatogastroenterology.

[45] Sategna-Guidetti C (1998). Autoimmune thyroid diseases and coeliac disease. Eur. J. Gastroenterol. Hepatol..

[46] Quatela A, Callister R, Patterson AJ, McEvoy M, MacDonald-Wicks LK (2017). Breakfast cereal consumption and obesity risk amongst the mid-age cohort of the Australian Longitudinal Study on Women's Health. Healthcare (Basel, Switzerland).

[47] Boskou D (2017). Edible cold pressed oils and their biologically active components. J. Exp. Food Chem..

[48] Desai ID, Bhagavan H, Salkeld R, DutradeOliveira JE (1988). Vitamin E content of crude and refined vegetable oils in Southern Brazil. J. Food Compos. Anal..

[49] Aparicio-Soto M (2017). The phenolic fraction of extra virgin olive oil modulates the activation and the inflammatory response of T cells from patients with systemic lupus erythematosus and healthy donors. Mol. Nutr. Food. Res..

[50] Kremer JM (1990). Dietary fish oil and olive oil supplementation in patients with rheumatoid arthritis. Clinical and immunologic effects. Arthritis Rheumat..

[51] Beauchamp GK (2005). Phytochemistry: Ibuprofen-like activity in extra-virgin olive oil. Nature.

[52] Perez-Jimenez F (2005). International conference on the healthy effect of virgin olive oil. Eur. J. Clin. Invest..

[53] Toledo E (2013). Effect of the Mediterranean diet on blood pressure in the PREDIMED trial: Results from a randomized controlled trial. BMC Med..

[54] Covas MI, de la Torre R, Fito M (2015). Virgin olive oil: A key food for cardiovascular risk protection. Br. J. Nutr..

[55] Carle A (2012). Moderate alcohol consumption may protect against overt autoimmune hypothyroidism: A population-based case–control study. Eur. J. Endocrinol..

[56] Calder PC, Yaqoob P (2009). Omega-3 polyunsaturated fatty acids and human health outcomes. BioFactors (Oxford, England).

[57] Simopoulos AP (2002). Omega-3 fatty acids in inflammation and autoimmune diseases. J. Am. Coll. Nutr..

[58] Swanson D, Block R, Mousa SA (2012). Omega-3 fatty acids EPA and DHA: Health benefits throughout life. Adv. Nutr..

[59] Calder PC (2017). Omega-3 fatty acids and inflammatory processes: From molecules to man. Biochem. Soc. Trans..

[60] Benvenga S (2016). Type of fish consumed and thyroid autoimmunity in pregnancy and postpartum. Endocrine.

[61] Nutrition Research Council (1989). Recommended Dietary Allowances, 10th ed.

[62] Rayman MP (2019). Multiple nutritional factors and thyroid disease, with particular reference to autoimmune thyroid disease. Proc. Nutr. Soc..

[63] Ertek S, Cicero AF, Caglar O, Erdogan G (2010). Relationship between serum zinc levels, thyroid hormones and thyroid volume following successful iodine supplementation. Hormones (Athens).

[64] Joseph SV, Edirisinghe I, Burton-Freeman BM (2016). Fruit polyphenols: A review of anti-inflammatory effects in humans. Crit. Rev. Food Sci. Nutr..

[65] Prehrambene smjernice za odrasle. *Hrvatski zavod za javno zdravstvo, Akademija medicinskih znanosti Hrvatske* (2002 godina.).

[66] U.S. Department of Health and Human Services and U.S. Department of Agriculture. *2015–2020 Dietary Guidelines for Americans*, 8th ed (2015).

[67] Kotsis V (2007). Hypertension and hypothyroidism: Results from an ambulatory blood pressure monitoring study. J. Hypertens..

[68] Perona JS (2004). Virgin olive oil reduces blood pressure in hypertensive elderly subjects. Clin. Nutr. (Edinburgh, Scotland).

[69] Martin-Pelaez S (2017). Effect of olive oil phenolic compounds on the expression of blood pressure-related genes in healthy individuals. Eur. J. Nutr..

[70] Tse Y (2017). Treatment algorithm for chronic idiopathic constipation and constipation-predominant irritable bowel syndrome derived from a Canadian National Survey and needs assessment on choices of therapeutic agents. Can. J. Gastroenterol. Hepatol..

[71] Venancio VP (2018). Polyphenol-rich Mango (*Mangifera indica* L.) Ameliorate functional constipation symptoms in humans beyond equivalent amount of fiber. Mol. Nutr. Food Res..

[72] Kilkkinen A (2001). Determinants of serum enterolactone concentration. Am. J. Clin. Nutr..

[73] Levin KA (2006). Study design III: Cross-sectional studies. Evid. Based Dent..

