# Supplementary material for: Differences in food consumption between patients with Hashimoto’s thyroiditis and healthy individuals
Source: Sci Rep. 2020 Jun 30;10:10670. doi: 10.1038/s41598-020-67719-7 (PMC7327046; doi:10.1038/s41598-020-67719-7)
Supplement: Supplementary file 1 — Supplementary information [file 41598_2020_67719_MOESM1_ESM.pdf]

Differences in food consumption between patients with Hashimoto's thyroiditis and healthy individuals

Dean Kaličanin[1], Luka Brčić[1], Katija Ljubetić[2], Ana Barić[3], Sanda Gračan[3], Marko Brekalo[3], Vesela Torlak Lovrić[3], Ivana Kolčić[4], Ozren Polašek[4], Tatijana Zemunik[1], Ante Punda[3], Vesna Boraska Perica[1]*

[1]University of Split, School of Medicine, Department of Medical Biology, 21000 Split, Croatia

[2]University of Rijeka, Faculty of Health Studies, Department of Clinical Nutrition, 51000 Rijeka

[3]University Hospital Split, Department of Nuclear Medicine, 21000 Split, Croatia

[4]University of Split, School of Medicine, Department of Public Health, 21000 Split, Croatia

Corresponding author: Vesna Boraska Perica, Assoc. Prof, University of Split, School of Medicine, Soltanska 2, 21 000 Split, Croatia
Phone: +385 21 557 873

E-mail: vboraska@mefst.hr

Supplementary Table 1. The list of 22 food groups formed from 48 food items from FFQ

MILK and MILK PRODUCTS: Milk, Yoghurt, Sour cream, Cottage cheese, Melted cheese, Hard cheese
RED MEAT: Pork, Veal, Beef, Lamb, Internal organs – liver, brains,lungs, etc.
PROCESEED MEAT: Bacon, Sausages, Salami
WHITE MEAT: Chicken, Turkey
WHITE FISH: White fish
OILY FISH: Big and small (salted pilchard) oily fish
SEA FOOD: Sea-food (shells, crab), Squid and Octopus
VEGETABLES: Leafy vegetables (cabbage, kale), Root vegetables (carrot, beet root), Flower vegetables (broccoli), Fruity vegetables (tomato), Legumes, Canned and pickled vegetables
EGGS: Eggs
POTATO: Potato
FRUITS: Fresh fruits, Dried fruit
NUTS: Nuts
REFINED GRAINS: White bread, Macaroni or Rice
WHOLE GRAINS: Whole grain bread
SWEETS: Cakes, Chocolate, Cookies, Bonbons
JAM, MARMALADE: Jam and Marmelade
NON-ALCOHOLIC BEVERAGES: Cedevita (fruity flavoured vitamin instant drink), Soda
LIQUOR: Liquor
COFFEE and TEA: Coffee, Tea
PLANT OIL: Plant oil
OLIVE OIL: Olive oil
ANIMAL FAT: Animal fat

Supplementary Table 2. Calculation of weekly intake of food items for each frequency category from FFQ

Frequency categories	HT cases	Controls
	Weekly intake	
Every day	7	7
2-3 times a week	2.5	2.5
Once a week	1	1
Once a month	0.25	na
Rarely	0.125*	0.167**
Never	0	0
Always	7	
Sometimes	1	
Never	0	

*once in two months; **once in 6 weeks

Supplementary Table 3. Calculation of physical activity in HT patients

Physical activity score during every day's work			
Sitting	Light	Moderate	Hard
0	30	60	120

	Score of engagement in any sport activity			
	Daily	2-3 times a week	Once a week	Occasionally
Less than an hour	30	10	4	1
Between 1-2 hours	45	15	6	1.5
Over 2 hours	60	20	8	2

Physical activity was assessed during the every day's work (with four responses: sitting, light, moderate and hard) and engagement in any sport activity (with four responses occasionally, once a week, 2-3 times a week and daily). Physical activity was calculated as a sum of physical activity score during every day's work and score of engagement in any sport activity for each HT patient.

Supplementary Table 4. Comparison of weekly intake frequency for 22 food groups between HT cases and controls

Food groups	Mean (SD)* HT cases	Mean (SD) controls	OR (95% CI)**	P value
More frequently consumed in HT cases than in controls				
**ANIMAL FAT**	0.9 (1.5)	0.5 (1.2)	1.55 (1.30-1.86)	**<0.0001**
**PROCESSED MEAT**	2.4 (2.4)	2.1 (2.5)	1.16 (1.06-1.27)	**0.0012**
**NUTS**	1.5 (1.9)	1.2 (1.7)	1.23 (1.08-1.40)	**0.0015**
POTATOES	2.9 (2.1)	2.8 (2.0)	1.11 (1.00-1.23)	0.0545
JAM, MARMALADE	1.1 (1.6)	1.1 (1.7)	1.08 (0.96-1.21)	0.2252
EGGS	1.6 (1.3)	1.2 (1.2)	1.08 (0.92-1.27)	0.3724
SWEETS	5.9 (5.3)	5.6 (5.3)	1.001 (0.96-1.04)	0.9576
Less frequently consumed in HT cases than in controls				
**RED MEAT**	2.5 (2.0)	3.5 (2.6)	0.80 (0.72-0.89)	**<0.0001**
**NON-ALCOHOLIC BEVERAGES**	1.5 (2.3)	2.4 (2.9)	0.82 (0.75-0.89)	**<0.0001**
**WHOLE GRAINS**	1.8 (2.2)	2.8 (2.8)	0.82 (0.76-0.89)	**<0.0001**
**PLANT OIL**	3.8 (3.1)	4.7 (3.0)	0.87 (0.82-0.93)	**<0.0001**
**OLIVE OIL**	3.6 (3.0)	4.6 (3.0)	0.90 (0.84-0.96)	**0.0018**
**LIQUOR**	0.1 (0.3)	0.3 (0.9)	0.49 (0.29-0.84)	**0.0087**
**OILY FISH**	0.8 (0.9)	1.2 (1.1)	0.72 (0.54-0.96)	**0.0245**
**FRUITS**	5.2 (3.2)	5.6 (3.1)	0.93 (0.87-0.996)	**0.0389**
REFINED GRAINS	6.2 (3.7)	6.5 (3.5)	0.96 (0.90-1.02)	0.1437
MILK and MILK PRODUCTS	10.1 (5.6)	11.0 (6.0)	0.98 (0.95-1.02)	0.3077
COFFEE and TEA	7.9 (3.9)	8.4 (3.9)	0.98 (0.93-1.03)	0.4120
WHITE FISH	0.7 (0.9)	1.0 (1.0)	0.91 (0.69-1.20)	0.4946
VEGETABLES	9.8 (6.1)	10.8 (6.2)	0.99 (0.96-1.03)	0.6614
WHITE MEAT	2.8 (1.9)	2.8 (2.0)	0.99 (0.89-1.09)	0.7794
SEAFOOD	0.6 (1.1)	0.8 (1.1)	0.99 (0.77-1.29)	0.9660

Food groups are ordered from the lowest to the highest *p* value. Food groups with the same *p* value (<0.0001) are ordered from the lowest to the highest OR. *p* <0.05 are shown in bold.
*SD- standard deviation, **OR (95% CI)- odds ratio with 95% confidence interval

Supplementary Table 5. Comparison of weekly intake of 22 food groups between 177 HT patients who were on LT4 therapy and 289 HT patients without LT4 therapy

Food groups	Mean (SD)* HT patients on therapy	Mean (SD) HT patients without therapy	OR (95% CI)*	P value
**RED MEAT**	2.9 (2.2)	2.3 (1.9)	1.24 (1.08-1.43)	**0.003**
LIQUOR	0.2 (0.3)	0.1 (0.2)	2.14 (0.96-4.79)	0.065
NON-ALCOHOLIC BEVERAGES	1.7 (2.6)	1.3 (2.0)	1.10 (0.98-1.22)	0.110
ANIMAL FAT	1.1 (1.8)	0.8 (1.2)	1.11 (0.95-1.30)	0.173
OLIVE OIL	3.9 (3.1)	3.4 (3.0)	1.06 (0.97-1.14)	0.196
COFFEE and TEA	7.9 (3.8)	8.1 (4.1)	0.97 (0.91-1.03)	0.282
WHOLE GRAINS	1.9 (2.3)	1.7 (2.2)	1.06 (0.95-1.17)	0.287
PROCESEED MEAT	2.4 (2.4)	2.4 (2.3)	0.95 (0.86-1.06)	0.374
FRUITS	5.3 (3.3)	5.1 (3.1)	1.03 (0.95-1.11)	0.481
VEGETABLES	9.9 (6.2)	9.9 (6.2)	0.99 (0.95-1.03)	0.510
SWEETS	5.7 (5.2)	6.1 (5.4)	0.99 (0.94-1.03)	0.530
WHITE MEAT	2.8 (2.1)	2.8 (1.9)	1.04 (0.92-1.16)	0.558
JAM, MARMALADE	1.2 (1.8)	1.1 (1.6)	0.96 (0.84-1.11)	0.617
EGGS	1.6 (1.3)	1.6 (1.3)	1.04 (0.87-1.24)	0.653
WHITEFISH	0.8 (0.8)	0.7 (0.9)	1.08 (0.76-1.53)	0.667
PLANT OIL	3.8 (3.1)	3.9 (3.1)	1.01 (0.94-1.09)	0.748
POTATOES	3.0 (2.2)	2.9 (2.1)	0.98 (0.87-1.11)	0.757
SEAFOOD	0.6 (1.3)	0.6 (1.1)	1.05 (0.75-1.46)	0.778
OILY FISH	0.8 (1.1)	0.7 (0.7)	0.96 (0.65-1.43)	0.850
MILK and MILK PRODUCTS	10 (5.1)	10.3 (5.9)	0.998 (0.96-1.04)	0.916
REFINED GRAINS	6.3 (3.7)	6.2 (3.6)	1.002 (0.94-1.07)	0.961
NUTS	1.5 (2.0)	1.5 (1.9)	1.00 (0.87-1.15)	0.999

Information on LT4 therapy was not available for 25 HT patients. We used logistic regression model, where therapy status (with/without LT4 therapy) was used as dependent variable and 22 food groups as independent variables along with age and gender.

P <0.05 are shown in bold. LT4 - levothyroxine, SD*- standard deviation, OR (95% CI)* - odds ratio with 95% confidence interval

Supplementary Table 6. Association of weekly intake of 22 food groups with HT-related quantitative traits in HT patients

Phenotype	TSH								
	ALL			OT			NT		
Food groups	β	SE	P value	β	SE	P value	β	SE	P value
MILK and MILK PRODUCTS	0.002	0.010	0.8170	0.014	0.019	0.4570	-0.010	0.013	0.4500
RED MEAT	-0.027	0.034	0.4220	-0.009	0.052	0.8630	-0.014	0.049	0.7680
PROCESEED MEAT	0.000	0.025	0.9850	0.002	0.047	0.9620	-0.013	0.034	0.7110
WHITE MEAT	-0.006	0.031	0.8560	0.027	0.045	0.5510	-0.055	0.047	0.2480
WHITEFISH	-0.035	0.085	0.6760	0.029	0.151	0.8480	-0.002	0.112	0.9880
OILY FISH	-0.072	0.094	0.4460	-0.085	0.141	0.5450	-0.121	0.141	0.3910
SEAFOOD	0.062	0.073	0.3950	-0.023	0.106	0.8270	0.185	0.136	0.1750
VEGETABLES	0.012	0.010	0.2090	0.008	0.016	0.6290	0.008	0.013	0.5400
EGGS	0.032	0.041	0.4390	0.046	0.069	0.5040	-0.002	0.056	0.9780
POTATOES	0.006	0.030	0.8340	0.006	0.047	0.9040	0.033	0.041	0.4270
FRUITS	0.012	0.019	0.5270	0.029	0.027	0.3020	0.004	0.027	0.8770
NUTS	-0.046	0.031	0.1440	-0.124	0.051	0.0170	0.014	0.044	0.7540
REFINED GRAINS	0.023	0.016	0.1460	0.031	0.024	0.2090	0.014	0.022	0.5420
WHOLE GRAINS	-0.036	0.024	0.1300	-0.038	0.037	0.3130	-0.049	0.035	0.1650
SWEETS	0.003	0.011	0.8050	0.009	0.019	0.6280	-0.003	0.015	0.8320
JAM, MARMALADE	-0.026	0.035	0.4510	-0.019	0.054	0.7270	-0.017	0.049	0.7240
NON-ALCOHOLIC BEVERAGES	0.032	0.026	0.2210	0.028	0.037	0.4550	0.034	0.041	0.4030
LIQUOR	0.166	0.186	0.3730	0.248	0.263	0.3470	0.155	0.308	0.6150
COFFEE and TEA	0.004	0.014	0.7810	-0.009	0.024	0.7190	0.009	0.019	0.6450
PLANT OIL	-0.038	0.018	0.0380	-0.054	0.029	0.0640	-0.033	0.025	0.1870
OLIVE OIL	0.005	0.020	0.7890	0.029	0.033	0.3750	-0.011	0.026	0.6870
ANIMAL FAT	-0.065	0.035	0.0690	-0.081	0.048	0.0920	-0.048	0.057	0.3960

Food groups \ Phenotype	T3								
	ALL			OT			NT		
	β	SE	P value	β	SE	P value	β	SE	P value
**MILK and MILK PRODUCTS**	-0.003	0.010	0.7970	0.017	0.020	0.4020	-0.001	0.013	0.9380
**RED MEAT**	0.025	0.034	0.4600	0.029	0.053	0.5920	0.005	0.048	0.9180
**PROCESEED MEAT**	-0.002	0.025	0.9410	-0.009	0.048	0.8580	-0.003	0.034	0.9200
**WHITE MEAT**	-0.024	0.029	0.3990	-0.129	0.046	0.0060	0.045	0.041	0.2810
**WHITEFISH**	0.047	0.085	0.5780	0.190	0.155	0.2220	-0.026	0.108	0.8090
**OILY FISH**	-0.088	0.095	0.3560	-0.018	0.142	0.9000	-0.044	0.137	0.7480
**SEAFOOD**	0.084	0.073	0.2520	0.043	0.109	0.6960	0.087	0.134	0.5150
**VEGETABLES**	0.003	0.010	0.7550	0.008	0.016	0.6050	-0.005	0.013	0.6900
**EGGS**	0.030	0.042	0.4810	-0.007	0.071	0.9250	0.023	0.056	0.6820
**POTATOES**	-0.009	0.029	0.7510	-0.021	0.048	0.6580	-0.038	0.039	0.3280
**FRUITS**	-0.007	0.019	0.7190	0.001	0.028	0.9640	-0.012	0.026	0.6350
**NUTS**	-0.009	0.032	0.7720	0.035	0.052	0.5020	-0.040	0.043	0.3530
**REFINED GRAINS**	0.027	0.016	0.1000	-0.020	0.025	0.4300	0.059	0.022	0.0080
**WHOLE GRAINS**	0.035	0.024	0.1480	0.049	0.038	0.2060	0.045	0.035	0.2020
**SWEETS**	-0.004	0.011	0.6880	0.019	0.020	0.3340	-0.013	0.014	0.3530
**JAM, MARMALADE**	-0.009	0.035	0.7950	-0.073	0.056	0.1980	0.034	0.048	0.4830
**NON-ALCOHOLIC BEVERAGES**	0.001	0.027	0.9750	-0.021	0.038	0.5760	0.032	0.041	0.4290
**LIQUOR**	-0.230	0.188	0.2210	0.027	0.271	0.9210	-0.632	0.298	0.0350
**COFFEE and TEA**	0.007	0.014	0.6410	-0.003	0.025	0.8920	0.019	0.019	0.3190
**PLANT OIL**	0.069	0.018	<0.0001	0.102	0.030	0.0010	0.056	0.024	0.0220
**OLIVE OIL**	0.029	0.020	0.1520	0.021	0.034	0.5340	0.027	0.026	0.3050
**ANIMAL FAT**	0.023	0.036	0.5370	0.015	0.049	0.7540	0.056	0.057	0.3250

Food groups \ Phenotype	T4								
	ALL			OT			NT		
	β	SE	P value	β	SE	P value	β	SE	P value
**MILK and MILK PRODUCTS**	-0.012	0.010	0.2360	-0.003	0.022	0.8930	-0.007	0.012	0.5490
**RED MEAT**	0.019	0.034	0.5640	0.037	0.058	0.5270	-0.027	0.045	0.5470
**PROCESEED MEAT**	-0.014	0.025	0.5650	-0.037	0.052	0.4780	0.021	0.032	0.5140
**WHITE MEAT**	0.015	0.028	0.6000	-0.058	0.050	0.2490	0.064	0.039	0.1000
**WHITEFISH**	-0.079	0.083	0.3460	-0.047	0.167	0.7800	-0.146	0.101	0.1530
**OILY FISH**	-0.148	0.093	0.1130	-0.194	0.154	0.2110	0.002	0.129	0.9890
**SEAFOOD**	0.077	0.072	0.2870	0.152	0.118	0.1990	0.076	0.125	0.5450
**VEGETABLES**	0.006	0.009	0.5090	0.019	0.017	0.2750	0.001	0.012	0.9440
**EGGS**	0.062	0.041	0.1340	0.051	0.077	0.5070	0.059	0.053	0.2600
**POTATOES**	0.015	0.029	0.6040	-0.036	0.052	0.4870	0.039	0.037	0.2890
**FRUITS**	0.026	0.018	0.1520	0.012	0.031	0.6990	0.026	0.024	0.2780
**NUTS**	-0.007	0.031	0.8140	0.069	0.056	0.2260	-0.062	0.041	0.1260
**REFINED GRAINS**	0.023	0.016	0.1480	0.018	0.027	0.5180	0.024	0.020	0.2340
**WHOLE GRAINS**	0.015	0.024	0.5360	-0.002	0.042	0.9590	0.035	0.033	0.2850
**SWEETS**	-0.005	0.011	0.6420	-0.005	0.022	0.8010	0.006	0.013	0.6520
**JAM, MARMALADE**	-0.027	0.034	0.4400	-0.046	0.061	0.4530	-0.028	0.045	0.5280
**NON-ALCOHOLIC BEVERAGES**	0.037	0.026	0.1630	0.013	0.041	0.7560	0.048	0.038	0.2160
**LIQUOR**	-0.374	0.184	0.0420	-0.350	0.293	0.2350	-0.521	0.280	0.0640
**COFFEE and TEA**	0.023	0.014	0.1090	0.037	0.027	0.1690	0.023	0.018	0.1980
**PLANT OIL**	0.040	0.018	0.0270	0.089	0.032	0.0060	0.003	0.023	0.8920
**OLIVE OIL**	0.032	0.020	0.1010	0.013	0.036	0.7220	0.039	0.024	0.1120
**ANIMAL FAT**	-0.010	0.036	0.7730	0.020	0.053	0.7040	-0.044	0.054	0.4080

Food groups \ Phenotype	fT4								
	ALL			OT			NT		
	β	SE	P value	β	SE	P value	β	SE	P value
MILK and MILK PRODUCTS	0.001	0.011	0.9010	-0.003	0.020	0.8780	0.007	0.013	0.6050
RED MEAT	-0.002	0.035	0.9630	0.042	0.053	0.4320	-0.036	0.049	0.4650
PROCESEED MEAT	-0.025	0.026	0.3350	-0.077	0.048	0.1150	0.004	0.034	0.9130
WHITE MEAT	-0.009	0.032	0.7850	-0.011	0.047	0.8150	0.043	0.047	0.3630
WHITEFISH	-0.018	0.086	0.8350	-0.125	0.155	0.4240	-0.019	0.111	0.8670
OILY FISH	0.003	0.096	0.9710	-0.052	0.145	0.7230	0.131	0.140	0.3510
SEAFOOD	-0.016	0.074	0.8300	0.099	0.109	0.3660	-0.084	0.138	0.5420
VEGETABLES	0.001	0.010	0.8980	0.004	0.016	0.8150	-0.001	0.013	0.9120
EGGS	-0.080	0.042	0.0580	-0.127	0.071	0.0780	-0.041	0.056	0.4600
POTATOES	0.011	0.030	0.7210	0.048	0.048	0.3250	-0.013	0.041	0.7430
FRUITS	0.005	0.019	0.8000	0.026	0.028	0.3680	-0.013	0.027	0.6310
NUTS	0.012	0.032	0.7170	0.033	0.053	0.5370	-0.008	0.044	0.8560
REFINED GRAINS	0.006	0.016	0.7110	0.013	0.025	0.6100	0.003	0.022	0.9090
WHOLE GRAINS	0.022	0.024	0.3550	0.037	0.039	0.3460	0.019	0.035	0.5770
SWEETS	0.002	0.011	0.8530	0.012	0.020	0.5510	0.010	0.015	0.4860
JAM, MARMALADE	-0.009	0.036	0.8100	-0.048	0.056	0.3870	-0.004	0.049	0.9290
NON-ALCOHOLIC BEVERAGES	-0.012	0.027	0.6590	-0.019	0.038	0.6130	-0.044	0.041	0.2840
LIQUOR	0.003	0.188	0.9890	0.216	0.270	0.4260	-0.138	0.305	0.6520
COFFEE and TEA	0.000	0.015	0.9640	-0.008	0.025	0.7340	0.006	0.019	0.7580
PLANT OIL	0.020	0.018	0.2710	0.028	0.030	0.3530	0.012	0.024	0.6280
OLIVE OIL	0.011	0.020	0.6040	0.005	0.034	0.8860	0.025	0.026	0.3480
ANIMAL FAT	0.030	0.036	0.4010	0.064	0.049	0.1960	-0.007	0.057	0.9000

Food groups \ Phenotype	TgAb								
	ALL			OT			NT		
	β	SE	P value	β	SE	P value	β	SE	P value
**MILK and MILK PRODUCTS**	-0.008	0.011	0.4900	-0.015	0.021	0.4620	-0.008	0.014	0.5690
**RED MEAT**	-0.070	0.036	0.0520	-0.124	0.056	0.0280	-0.028	0.050	0.5790
**PROCESEED MEAT**	0.004	0.027	0.8810	0.060	0.051	0.2430	0.000	0.035	0.9880
**WHITE MEAT**	-0.061	0.030	0.0450	-0.049	0.049	0.3180	-0.079	0.043	0.0700
**WHITEFISH**	0.053	0.089	0.5540	-0.196	0.162	0.2310	0.162	0.113	0.1510
**OILY FISH**	-0.037	0.099	0.7080	0.054	0.150	0.7180	-0.133	0.144	0.3570
**SEAFOOD**	-0.022	0.077	0.7700	-0.007	0.114	0.9500	0.027	0.140	0.8450
**VEGETABLES**	0.016	0.010	0.1050	0.009	0.017	0.6090	0.018	0.014	0.1980
**EGGS**	-0.007	0.044	0.8660	0.064	0.075	0.3930	-0.065	0.059	0.2670
**POTATOES**	-0.010	0.031	0.7450	0.072	0.051	0.1580	-0.050	0.041	0.2250
**FRUITS**	0.022	0.020	0.2690	0.021	0.030	0.4740	0.025	0.027	0.3460
**NUTS**	0.017	0.033	0.6060	0.042	0.055	0.4490	0.006	0.045	0.8870
**REFINED GRAINS**	-0.013	0.017	0.4290	-0.008	0.026	0.7700	-0.011	0.023	0.6150
**WHOLE GRAINS**	-0.031	0.025	0.2230	0.010	0.040	0.8010	-0.048	0.036	0.1930
**SWEETS**	-0.017	0.012	0.1300	-0.047	0.021	0.0280	0.000	0.015	0.9860
**JAM, MARMALADE**	0.034	0.037	0.3580	0.065	0.059	0.2750	0.032	0.050	0.5270
**NON-ALCOHOLIC BEVERAGES**	0.005	0.028	0.8490	0.001	0.040	0.9740	-0.020	0.043	0.6380
**LIQUOR**	0.131	0.196	0.5030	-0.115	0.284	0.6880	0.133	0.311	0.6680
**COFFEE and TEA**	-0.017	0.015	0.2690	-0.015	0.026	0.5760	-0.013	0.020	0.5090
**PLANT OIL**	0.022	0.019	0.2520	-0.002	0.031	0.9560	0.029	0.025	0.2510
**OLIVE OIL**	0.012	0.021	0.5600	-0.015	0.035	0.6780	0.036	0.027	0.1840
**ANIMAL FAT**	-0.029	0.038	0.4520	-0.077	0.051	0.1350	0.010	0.060	0.8730

Food groups \ Phenotype	TPOAb								
	ALL			OT			NT		
	β	SE	P value	β	SE	P value	β	SE	P value
**MILK and MILK PRODUCTS**	0.007	0.011	0.4930	0.013	0.019	0.5040	0.003	0.014	0.8560
**RED MEAT**	-0.067	0.035	0.0550	-0.066	0.052	0.2050	-0.085	0.050	0.0900
**PROCESEED MEAT**	-0.012	0.026	0.6370	0.010	0.047	0.8290	0.004	0.035	0.9120
**WHITE MEAT**	-0.043	0.029	0.1420	-0.050	0.045	0.2740	-0.065	0.043	0.1330
**WHITEFISH**	0.154	0.086	0.0750	0.203	0.151	0.1820	0.209	0.113	0.0650
**OILY FISH**	-0.049	0.096	0.6090	-0.104	0.139	0.4560	-0.104	0.144	0.4700
**SEAFOOD**	0.024	0.074	0.7460	0.034	0.106	0.7510	0.102	0.140	0.4680
**VEGETABLES**	-0.002	0.010	0.8690	-0.004	0.016	0.8050	-0.005	0.014	0.7090
**EGGS**	0.027	0.042	0.5300	0.149	0.070	0.0350	-0.049	0.059	0.4020
**POTATOES**	0.028	0.030	0.3490	0.026	0.047	0.5810	0.022	0.041	0.5900
**FRUITS**	-0.008	0.019	0.6580	-0.018	0.028	0.5070	0.006	0.027	0.8210
**NUTS**	0.012	0.032	0.7110	-0.005	0.051	0.9230	0.014	0.045	0.7590
**REFINED GRAINS**	-0.027	0.016	0.1020	-0.043	0.025	0.0800	-0.020	0.023	0.3870
**WHOLE GRAINS**	-0.057	0.025	0.0220	-0.028	0.038	0.4620	-0.086	0.036	0.0190
**SWEETS**	-0.011	0.011	0.3410	0.000	0.019	0.9790	-0.008	0.015	0.5940
**JAM, MARMALADE**	-0.045	0.036	0.2100	-0.118	0.055	0.0330	0.002	0.050	0.9750
**NON-ALCOHOLIC BEVERAGES**	0.000	0.027	0.9900	0.004	0.037	0.9070	-0.001	0.043	0.9790
**LIQUOR**	0.039	0.190	0.8390	-0.235	0.265	0.3760	0.298	0.311	0.3390
**COFFEE and TEA**	-0.007	0.015	0.6150	0.020	0.024	0.4090	-0.008	0.020	0.6950
**PLANT OIL**	0.023	0.019	0.2140	0.034	0.029	0.2480	0.009	0.025	0.7110
**OLIVE OIL**	-0.001	0.020	0.9550	-0.007	0.033	0.8260	-0.002	0.027	0.9320
**ANIMAL FAT**	0.046	0.037	0.2180	0.088	0.048	0.0670	-0.006	0.060	0.9210

Phenotype / Food groups	Thyroid volume								
	ALL			OT			NT		
	β	SE	P value	β	SE	P value	β	SE	P value
MILK and MILK PRODUCTS	0.004	0.011	0.6720	0.022	0.024	0.3620	0.006	0.011	0.5990
RED MEAT	0.007	0.035	0.8460	0.013	0.065	0.8400	-0.031	0.042	0.4660
PROCESEED MEAT	-0.007	0.026	0.7800	-0.041	0.061	0.5000	-0.016	0.029	0.5950
WHITE MEAT	-0.040	0.031	0.1930	-0.013	0.055	0.8100	-0.072	0.040	0.0750
WHITEFISH	-0.127	0.084	0.1320	-0.112	0.189	0.5520	-0.077	0.093	0.4120
OILY FISH	-0.042	0.093	0.6560	-0.328	0.173	0.0610	-0.024	0.118	0.8360
SEAFOOD	0.040	0.074	0.5870	0.246	0.140	0.0820	-0.140	0.116	0.2280
VEGETABLES	0.016	0.010	0.1110	0.020	0.020	0.3270	0.018	0.011	0.1170
EGGS	0.037	0.041	0.3670	0.188	0.084	0.0280	-0.037	0.048	0.4430
POTATOES	-0.058	0.030	0.0530	-0.004	0.060	0.9450	-0.087	0.035	0.0130
FRUITS	0.009	0.019	0.6220	-0.006	0.034	0.8710	0.013	0.023	0.5580
NUTS	-0.008	0.032	0.8090	0.019	0.066	0.7700	-0.017	0.037	0.6540
REFINED GRAINS	0.011	0.016	0.4780	0.030	0.030	0.3220	0.011	0.019	0.5750
WHOLE GRAINS	-0.044	0.024	0.0700	-0.037	0.047	0.4310	-0.054	0.030	0.0720
SWEETS	0.011	0.011	0.3320	0.017	0.024	0.4990	0.010	0.012	0.4310
JAM, MARMALADE	-0.019	0.035	0.5890	-0.076	0.067	0.2590	0.020	0.041	0.6330
NON-ALCOHOLIC BEVERAGES	0.009	0.026	0.7280	0.008	0.046	0.8670	0.023	0.034	0.5100
LIQUOR	0.122	0.205	0.5540	-0.301	0.396	0.4490	0.289	0.256	0.2600
COFFEE and TEA	0.006	0.014	0.6720	0.042	0.029	0.1540	-0.007	0.016	0.6480
PLANT OIL	0.005	0.018	0.7680	-0.006	0.037	0.8650	0.017	0.021	0.4260
OLIVE OIL	0.021	0.020	0.3150	0.011	0.042	0.7850	0.026	0.023	0.2630
ANIMAL FAT	-0.069	0.036	0.0550	-0.029	0.061	0.6370	-0.064	0.048	0.1790

Food groups \ Phenotype	Systolic blood pressure								
	ALL			OT			NT		
	β	SE	P value	β	SE	P value	β	SE	P value
**MILK and MILK PRODUCTS**	0.005	0.012	0.6630	-0.014	0.026	0.582	0.014	0.014	0.3450
**RED MEAT**	0.031	0.042	0.4570	0.056	0.070	0.432	0.003	0.056	0.9640
**PROCESEED MEAT**	-0.021	0.028	0.4610	0.061	0.059	0.308	-0.050	0.036	0.1690
**WHITE MEAT**	0.013	0.034	0.7070	0.001	0.050	0.986	0.010	0.057	0.8620
**WHITEFISH**	-0.063	0.107	0.5540	-0.131	0.192	0.499	-0.021	0.140	0.8800
**OILY FISH**	0.138	0.107	0.2000	0.203	0.166	0.225	0.102	0.163	0.5300
**SEAFOOD**	-0.057	0.081	0.4790	-0.118	0.148	0.428	-0.060	0.160	0.7090
**VEGETABLES**	0.012	0.012	0.2980	0.048	0.022	0.030	-0.006	0.016	0.7290
**EGGS**	-0.045	0.054	0.4010	0.112	0.103	0.282	-0.151	0.071	0.0370
**POTATOES**	0.039	0.036	0.2810	-0.047	0.067	0.486	0.030	0.048	0.5320
**FRUITS**	-0.012	0.022	0.5750	-0.057	0.036	0.118	0.000	0.030	0.9940
**NUTS**	-0.013	0.039	0.7300	0.060	0.064	0.355	-0.044	0.054	0.4180
**REFINED GRAINS**	0.019	0.019	0.3060	-0.040	0.033	0.221	0.050	0.025	0.0500
**WHOLE GRAINS**	-0.007	0.027	0.8070	-0.013	0.046	0.775	0.028	0.041	0.4890
**SWEETS**	0.005	0.012	0.6850	0.004	0.022	0.846	0.002	0.016	0.9100
**JAM, MARMALADE**	-0.053	0.043	0.2180	-0.071	0.068	0.305	0.021	0.061	0.7340
**NON-ALCOHOLIC BEVERAGES**	-0.021	0.029	0.4750	0.054	0.048	0.260	-0.055	0.044	0.2110
**LIQUOR**	-0.068	0.187	0.7160	-0.226	0.278	0.419	-0.008	0.310	0.9790
**COFFEE and TEA**	0.003	0.017	0.8760	0.000	0.030	0.986	0.025	0.023	0.2730
**PLANT OIL**	-0.009	0.022	0.6700	-0.006	0.038	0.879	-0.024	0.028	0.3870
**OLIVE OIL**	-0.031	0.024	0.1870	-0.159	0.047	0.001	-0.017	0.031	0.5760
**ANIMAL FAT**	-0.016	0.041	0.6940	-0.050	0.070	0.477	-0.018	0.056	0.7450

Phenotype / Food groups	Diastolic blood pressure								
	ALL			OT			NT		
	β	SE	P value	β	SE	P value	β	SE	P value
MILK and MILK PRODUCTS	0.011	0.012	0.3640	0.002	0.029	0.937	0.013	0.014	0.3650
RED MEAT	0.033	0.043	0.4450	0.101	0.078	0.199	0.015	0.057	0.7990
PROCESEED MEAT	-0.037	0.029	0.2060	0.036	0.065	0.583	-0.034	0.036	0.3540
WHITE MEAT	0.001	0.035	0.9700	0.032	0.056	0.563	-0.071	0.057	0.2140
WHITEFISH	-0.173	0.109	0.1150	-0.433	0.211	0.045	-0.063	0.139	0.6520
OILY FISH	0.030	0.109	0.7860	0.229	0.182	0.215	-0.079	0.161	0.6270
SEAFOOD	0.127	0.082	0.1250	-0.016	0.163	0.921	0.281	0.159	0.0800
VEGETABLES	-0.004	0.012	0.7170	0.020	0.024	0.391	-0.024	0.016	0.1280
EGGS	-0.008	0.055	0.8790	-0.075	0.114	0.512	-0.070	0.071	0.3280
POTATOES	0.085	0.037	0.0240	0.048	0.074	0.522	0.086	0.048	0.0740
FRUITS	0.000	0.022	0.9930	0.025	0.039	0.532	-0.015	0.029	0.6010
NUTS	-0.024	0.039	0.5440	0.068	0.071	0.341	-0.031	0.054	0.5700
REFINED GRAINS	-0.014	0.019	0.4790	-0.072	0.036	0.048	0.001	0.025	0.9650
WHOLE GRAINS	-0.025	0.028	0.3700	0.021	0.050	0.671	-0.041	0.042	0.3290
SWEETS	0.002	0.012	0.8980	-0.023	0.025	0.361	0.009	0.016	0.5860
JAM, MARMALADE	-0.073	0.044	0.0980	-0.070	0.075	0.355	-0.019	0.060	0.7550
NON-ALCOHOLIC BEVERAGES	-0.042	0.031	0.1720	0.024	0.052	0.643	-0.033	0.046	0.4770
LIQUOR	-0.145	0.191	0.4480	-0.156	0.306	0.613	0.065	0.308	0.8320
COFFEE and TEA	0.005	0.017	0.7910	-0.035	0.033	0.288	0.038	0.022	0.0930
PLANT OIL	-0.018	0.022	0.4170	-0.013	0.042	0.763	-0.041	0.027	0.1370
OLIVE OIL	-0.011	0.024	0.6620	-0.124	0.051	0.019	0.018	0.031	0.5620
ANIMAL FAT	-0.032	0.042	0.4410	-0.105	0.077	0.177	-0.046	0.055	0.4130

Analyses were performed in 3 groups: ALL - entire set of 491 HT patients; OT - 177 HT patients on LT4 therapy; NT - 289 HT patients without LT4 therapy. For 25 HT patients information on LT4 therapy was not available. Statistically significant values (P<0.0055) are dark grey shaded, nominally significant values (0.01<P>0.005) are light grey shaded. Linear regression model was used, where selected quantitative trait was dependent variable and 22 food groups were independent variables. Age, gender and physical activity were also included as covariates. For thyroid volume, systolic and diastolic blood pressure we used BSA and TSH as covariates, while for TSH and fT4, we used BSA as covariate. In the group of all HT patients we used LT4 therapy status as an additional covariate, whereas, in the sub-group of HT patients that were on LT4 therapy, we used LT4 therapy dose and weight ratio as additional covariate. β - effect size, SE - standard error.

Supplementary Table 7. Association of 22 food groups with 16 symptoms of hypothyroidism in 289 HT patients without therapy

Symptoms / Food groups	Weakness		Dry, rough skin		Slow speach		Edem of the eyes	
	**OR (95% CI)**	**P value**	**OR (95% CI)**	**P value**	**OR (95% CI)**	**P value**	**OR (95% CI)**	**P value**
**MILK and MILK PRODUCTS**	1.01 (0.94 - 1.08)	0.8500	0.998 (0.93 - 1.07)	0.9580	0.92 (0.77 - 1.10)	0.3470	1.00 (0.91 - 1.09)	0.9970
**RED MEAT**	1.05 (0.81 - 1.36)	0.7340	0.92 (0.7 - 1.22)	0.5760	1.23 (0.71 - 2.13)	0.4680	0.89 (0.61 - 1.29)	0.5320
**PROCESEED MEAT**	0.98 (0.81 - 1.17)	0.7840	1.10 (0.92 - 1.32)	0.2940	1.17 (0.77 - 1.78)	0.4680	0.93 (0.73 - 1.19)	0.5620
**WHITE MEAT**	1.09 (0.83 - 1.43)	0.5230	1.40 (1.04 - 1.89)	0.0260	0.88 (0.40 - 1.94)	0.7560	1.39 (0.96 - 2.01)	0.0860
**WHITEFISH**	0.79 (0.47 - 1.33)	0.3660	1.22 (0.70 - 2.10)	0.4860	1.21 (0.33 - 4.45)	0.7720	1.15 (0.62 - 2.16)	0.6570
**OILY FISH**	1.14 (0.55 - 2.36)	0.7260	1.08 (0.50 - 2.31)	0.8450	1.33 (0.20 - 8.83)	0.7690	2.01 (0.75 - 5.40)	0.1660
**SEAFOOD**	1.14 (0.56 - 2.31)	0.7170	1.10 (0.55 - 2.18)	0.7890	0.80 (0.15 - 4.13)	0.7860	0.81 (0.34 - 1.92)	0.6330
**VEGETABLES**	0.96 (0.89 - 1.03)	0.2620	0.94 (0.87 - 1.02)	0.1340	0.98 (0.81 - 1.18)	0.8250	1.01 (0.92 - 1.11)	0.8170
**EGGS**	1.13 (0.84 - 1.53)	0.4200	0.98 (0.71 - 1.33)	0.8740	1.11 (0.29 - 4.23)	0.8740	0.86 (0.55 - 1.34)	0.5050
**POTATOES**	0.95 (0.76 - 1.19)	0.6630	0.89 (0.69 - 1.15)	0.3760	1.03 (0.56 - 1.90)	0.9150	1.05 (0.74 - 1.48)	0.7980
**FRUITS**	0.995 (0.86 - 1.15)	0.9440	1.09 (0.94 - 1.26)	0.2420	0.99 (0.68 - 1.42)	0.9410	0.80 (0.64 - 0.98)	0.0350
**NUTS**	0.87 (0.70 - 1.10)	0.2390	1.17 (0.92 - 1.47)	0.2020	0.98 (0.55 - 1.75)	0.9410	0.96 (0.71 - 1.30)	0.7950
**REFINED GRAINS**	0.93 (0.83 - 1.05)	0.2500	1.08 (0.95 - 1.22)	0.2470	1.02 (0.74 - 1.41)	0.8880	0.96 (0.81 - 1.13)	0.6090
**WHOLE GRAINS**	0.96 (0.80 - 1.14)	0.6130	0.98 (0.80 - 1.20)	0.8310	0.84 (0.54 - 1.30)	0.4250	1.09 (0.86 - 1.37)	0.4890
**SWEETS**	0.95 (0.88 - 1.03)	0.1930	0.95 (0.87 - 1.04)	0.2290	0.71 (0.45 - 1.13)	0.1430	0.91 (0.79 - 1.03)	0.1400
**JAM, MARMALADE**	1.14 (0.86 - 1.52)	0.3670	1.11 (0.83 - 1.49)	0.4890	1.11 (0.52 - 2.34)	0.7920	0.89 (0.57 - 1.37)	0.5930
**NON-ALCOHOLIC BEVERAGES**	0.92 (0.74 - 1.16)	0.4880	1.08 (0.86 - 1.37)	0.5060	1.65 (0.95 - 2.87)	0.0770	1.10 (0.79 - 1.54)	0.5610
**LIQUOR**	0.84 (0.15 - 4.73)	0.8460	1.88 (0.42 - 8.36)	0.4090	0.97 (0.0- 6747)	0.9950	1.17 (0.2 - 6.75)	0.8600
**COFFEE and TEA**	1.03 (0.93 - 1.14)	0.6290	0.93 (0.84 - 1.02)	0.1320	0.999 (0.78 - 1.28)	0.9970	1.06 (0.92 - 1.21)	0.4270
**PLANT OIL**	1.03 (0.90 - 1.18)	0.6510	0.97 (0.85 - 1.11)	0.6310	0.87 (0.61 - 1.25)	0.4530	0.92 (0.76 - 1.11)	0.3650
**OLIVE OIL**	0.99 (0.85 - 1.14)	0.8560	1.18 (1.01 - 1.37)	0.0330	0.88 (0.63 - 1.24)	0.4660	1.04 (0.85 - 1.27)	0.7080
**ANIMAL FAT**	0.98 (0.73 - 1.32)	0.8790	1.08 (0.79 - 1.48)	0.6170	0.27 (0.04 - 1.90)	0.1890	1.32 (0.83 - 2.09)	0.2430

Food groups \ Symptoms	Sensitivity to coldness		Cold skin		Face edem		Fragile hair	
	OR (95% CI)	P value	OR (95% CI)	P value	OR (95% CI)	P value	OR (95% CI)	P value
**MILK and MILK PRODUCTS**	0.98 (0.92 - 1.05)	0.6290	1.01 (0.94 - 1.08)	0.8690	0.98 (0.86 - 1.11)	0.7040	0.95 (0.89 - 1.02)	0.1750
**RED MEAT**	0.89 (0.69 - 1.16)	0.4070	0.88 (0.66 - 1.17)	0.3640	1.31 (0.83 - 2.07)	0.2480	0.99 (0.74 - 1.31)	0.9330
**PROCESEED MEAT**	1.01 (0.85 - 1.19)	0.9360	0.99 (0.82 - 1.19)	0.8720	1.001 (0.73 - 1.37)	0.9950	0.999 (0.84 - 1.19)	0.9900
**WHITE MEAT**	1.10 (0.84 - 1.44)	0.4790	1.26 (0.92 - 1.70)	0.1450	0.95 (0.58 - 1.54)	0.8220	1.20 (0.89 - 1.63)	0.2310
**WHITEFISH**	0.91 (0.54 - 1.55)	0.7430	0.91 (0.45 - 1.83)	0.7830	1.37 (0.61 - 3.08)	0.4510	1.39 (0.69 - 2.81)	0.3550
**OILY FISH**	1.04 (0.50 - 2.18)	0.9200	1.08 (0.47 - 2.46)	0.8600	0.68 (0.20 - 2.35)	0.5380	0.74 (0.31 - 1.75)	0.4930
**SEAFOOD**	1.00 (0.50 - 1.20)	0.9990	1.55 (0.77 - 3.10)	0.2200	1.05 (0.36 - 3.03)	0.9340	1.54 (0.75 - 3.15)	0.2360
**VEGETABLES**	0.97 (0.90 - 1.04)	0.3900	0.97 (0.90 - 1.05)	0.5200	1.08 (0.97 - 1.22)	0.1720	1.02 (0.94 - 1.11)	0.5830
**EGGS**	0.89 (0.66 - 1.21)	0.4700	1.07 (0.77 - 1.47)	0.6990	0.94 (0.46 - 1.91)	0.8540	1.24 (0.91 - 1.71)	0.1800
**POTATOES**	1.10 (0.88 - 1.37)	0.4160	1.31 (1.03 - 1.67)	0.0260	1.44 (0.94 - 2.21)	0.0920	0.82 (0.62 - 1.08)	0.1530
**FRUITS**	1.07 (0.93 - 1.22)	0.3280	1.08 (0.93 - 1.26)	0.3380	1.10 (0.88 - 1.38)	0.3850	1.17 (1.02 - 1.36)	0.0310
**NUTS**	1.10 (0.88 - 1.39)	0.3970	1.20 (0.95 - 1.53)	0.1330	1.54 (1.06 - 2.24)	0.0220	1.05 (0.83 - 1.34)	0.6750
**REFINED GRAINS**	1.02 (0.91 - 1.15)	0.7290	0.94 (0.83 - 1.07)	0.3300	0.94 (0.75 - 1.17)	0.5700	1.05 (0.93 - 1.20)	0.4280
**WHOLE GRAINS**	1.03 (0.86 - 1.23)	0.7420	0.92 (0.75 - 1.14)	0.4560	0.85 (0.60 - 1.21)	0.3650	1.02 (0.84 - 1.24)	0.8420
**SWEETS**	0.97 (0.89 - 1.05)	0.4540	1.06 (0.97 - 1.15)	0.1930	0.85 (0.69 - 1.05)	0.1390	0.997 (0.91 - 1.09)	0.9460
**JAM, MARMALADE**	0.93 (0.68 - 1.27)	0.6440	0.93 (0.68 - 1.28)	0.6560	0.80 (0.36 - 1.79)	0.5850	0.89 (0.63 - 1.27)	0.5280
**NON-ALCOHOLIC BEVERAGES**	0.97 (0.77 - 1.22)	0.7800	1.05 (0.84 - 1.32)	0.6750	1.33 (0.87 - 2.03)	0.1890	1.17 (0.93 - 1.47)	0.1730
**LIQUOR**	0.29 (0.03 - 2.99)	0.3010	1.06 (0.19 - 5.81)	0.9500	1.02 (0.02 - 53.8)	0.9910	0.54 (0.06 - 4.56)	0.5690
**COFFEE and TEA**	1.09 (0.99 - 1.21)	0.0920	0.90 (0.81 - 1.00)	0.0500	0.96 (0.81 - 1.14)	0.6730	0.95 (0.86 - 1.05)	0.3130
**PLANT OIL**	0.97 (0.86 - 1.11)	0.6750	0.96 (0.84 - 1.10)	0.5650	1.09 (0.85 - 1.40)	0.5080	0.995 (0.86 - 1.14)	0.9390
**OLIVE OIL**	0.98 (0.85 - 1.13)	0.7840	1.07 (0.92 - 1.25)	0.3830	1.08 (0.82 - 1.40)	0.5950	0.95 (0.81 - 1.11)	0.4960
**ANIMAL FAT**	1.03 (0.75 - 1.42)	0.8580	1.08 (0.79 - 1.49)	0.6240	1.32 (0.81 - 2.14)	0.2600	1.08 (0.77 - 1.51)	0.6750

Symptoms	Pale skin		Memory disturbance		Constipation		Increase in weight	
**Food groups**	**OR (95% CI)**	**P value**	**OR (95% CI)**	**P value**	**OR (95% CI)**	**P value**	**OR (95% CI)**	**P value**
**MILK and MILK PRODUCTS**	1.01 (0.94 - 1.08)	0.8160	1.03 (0.96 - 1.11)	0.3870	1.02 (0.93 - 1.12)	0.6380	1.05 (0.97 - 1.13)	0.2390
**RED MEAT**	0.96 (0.73 - 1.27)	0.7920	0.71 (0.51 - 0.99)	0.0410	0.97 (0.68 - 1.40)	0.8760	0.96 (0.70 - 1.31)	0.8090
**PROCESEED MEAT**	0.93 (0.77 - 1.11)	0.4050	0.82 (0.65 - 1.03)	0.0890	0.84 (0.64 - 1.11)	0.2240	0.91 (0.74 - 1.12)	0.3680
**WHITE MEAT**	1.02 (0.77 - 1.35)	0.8940	1.02 (0.73 - 1.42)	0.9230	0.99 (0.64 - 1.54)	0.9560	0.96 (0.69 - 1.32)	0.7950
**WHITEFISH**	1.82 (0.97 - 3.40)	0.0620	0.33 (0.11 - 0.99)	0.0480	1.35 (0.65 - 2.82)	0.4240	1.22 (0.63 - 2.37)	0.5590
**OILY FISH**	0.62 (0.29 - 1.36)	0.2370	4.66 (1.52 - 14.2)	0.0070	1.69 (0.59 - 4.86)	0.3320	1.32 (0.54 - 3.19)	0.5440
**SEAFOOD**	0.94 (0.46 - 1.94)	0.8660	3.05 (1.13 - 8.22)	0.0280	0.88 (0.37 - 2.09)	0.7790	1.19 (0.55 - 2.59)	0.6520
**VEGETABLES**	0.97 (0.90 - 1.04)	0.3750	0.98 (0.89 - 1.08)	0.6260	0.99 (0.90 - 1.09)	0.8370	0.95 (0.87 - 1.03)	0.2230
**EGGS**	1.02 (0.73 - 1.41)	0.9210	0.58 (0.34 - 0.98)	0.0420	1.37 (0.91 - 2.04)	0.1290	1.007 (0.71 - 1.42)	0.9680
**POTATOES**	1.04 (0.82 - 1.31)	0.7540	1.15 (0.85 - 1.54)	0.3660	0.72 (0.51 - 1.03)	0.0720	1.07 (0.71 - 1.42)	0.6300
**FRUITS**	1.07 (0.93 - 1.23)	0.3740	1.06 (0.90 - 1.24)	0.5130	1.38 (1.12 - 1.70)	0.0020	0.98 (0.83 - 1.14)	0.7650
**NUTS**	0.99 (0.77 - 1.27)	0.9140	1.01 (0.76 - 1.32)	0.9720	0.94 (0.67 - 1.30)	0.6990	0.99 (0.76 - 1.30)	0.9680
**REFINED GRAINS**	0.99 (0.88 - 1.12)	0.8920	0.94 (0.80 - 1.10)	0.4240	0.997 (0.84 - 1.19)	0.9710	0.95 (0.83 - 1.10)	0.4700
**WHOLE GRAINS**	0.82 (0.66 - 1.01)	0.0560	0.88 (0.70 - 1.12)	0.3000	1.15 (0.90 - 1.47)	0.2490	0.94 (0.77 - 1.16)	0.5870
**SWEETS**	0.93 (0.85 - 1.02)	0.1060	0.88 (0.79 - 0.99)	0.0280	0.98 (0.87 - 1.10)	0.7400	1.04 (0.96 - 1.14)	0.3380
**JAM, MARMALADE**	1.01 (0.72 - 1.42)	0.9410	0.97 (0.64 - 1.47)	0.8860	1.10 (0.71 - 1.71)	0.6700	1.08 (0.75 - 1.55)	0.6740
**NON-ALCOHOLIC BEVERAGES**	1.10 (0.88 - 1.38)	0.4090	1.07 (0.83 - 1.39)	0.6050	1.05 (0.80 - 1.38)	0.7230	1.23 (0.97 - 1.57)	0.0860
**LIQUOR**	1.35 (0.22 - 8.43)	0.7500	4.11 (0.69 - 24.5)	0.1210	0.61 (0.01 - 30.4)	0.8020	0.63 (0.06 - 6.61)	0.7020
**COFFEE and TEA**	1.01 (0.91 - 1.12)	0.8880	0.91 (0.80 - 1.03)	0.1290	0.81 (0.70 - 0.94)	0.0040	1.04 (0.93 - 1.16)	0.4850
**PLANT OIL**	0.92 (0.81 - 1.06)	0.2480	1.08 (0.92 - 1.26)	0.3720	1.05 (0.87 - 1.26)	0.6330	0.95 (0.81 - 1.10)	0.4850
**OLIVE OIL**	0.99 (0.86 - 1.15)	0.9060	0.84 (0.70 - 1.01)	0.0570	0.86 (0.69 - 1.06)	0.1560	0.995 (0.84 - 1.18)	0.9570
**ANIMAL FAT**	1.08 (0.80 - 1.47)	0.6020	0.70 (0.41 - 1.20)	0.1980	1.21 (0.79 - 1.85)	0.3810	0.84 (0.51 - 1.38)	0.4830

Food groups \ Symptoms	Loss of skin hair		Dyspnea		Peripheral edems		Harsh voice	
	OR (95% CI)	P value	OR (95% CI)	P value	OR (95% CI)	P value	OR (95% CI)	P value
**MILK and MILK PRODUCTS**	1.21 (1.05 - 1.39)	0.0080	0.94 (0.82 - 1.08)	0.2840	0.94 (0.82 - 1.08)	0.3750	1.08 (0.99 - 1.18)	0.0860
**RED MEAT**	1.13 (0.72 - 1.77)	0.6040	0.82 (0.48 - 1.39)	0.5180	0.82 (0.48 - 1.39)	0.4560	0.88 (0.63 - 1.25)	0.4750
**PROCESEED MEAT**	1.15 (0.86 - 1.53)	0.3580	1.24 (0.90 - 1.70)	0.4560	1.24 (0.90 - 1.70)	0.1950	0.95 (0.74 - 1.21)	0.6610
**WHITE MEAT**	0.71 (0.42 - 1.20)	0.1980	0.94 (0.54 - 1.64)	0.0370	0.94 (0.54 - 1.64)	0.8180	1.21 (0.85 - 1.71)	0.2910
**WHITEFISH**	0.94 (0.39 - 2.26)	0.8880	0.39 (0.10 - 1.59)	0.0550	0.39 (0.10 - 1.59)	0.1900	0.92 (0.50 - 1.69)	0.7890
**OILY FISH**	0.87 (0.21 - 3.63)	0.8480	2.67 (0.80 - 8.94)	0.6710	2.67 (0.80 - 8.94)	0.1120	1.38 (0.50 - 3.79)	0.5350
**SEAFOOD**	0.71 (0.23 - 2.26)	0.5660	4.14 (1.29 - 13.3)	0.3290	4.14 (1.29 - 13.3)	0.0170	1.05 (0.45 - 2.44)	0.9180
**VEGETABLES**	1.03 (0.92 - 1.16)	0.5950	1.01 (0.88 - 1.16)	0.3270	1.01 (0.88 - 1.16)	0.8940	1.03 (0.94 - 1.13)	0.4780
**EGGS**	0.37 (0.14 - 0.98)	0.0440	0.83 (0.43 - 1.60)	0.7660	0.83 (0.43 - 1.60)	0.5810	0.81 (0.52 - 1.27)	0.3570
**POTATOES**	0.94 (0.59 - 1.49)	0.7830	1.57 (1.01 - 2.43)	0.5330	1.57 (1.01 - 2.43)	0.0460	1.07 (0.80 - 1.44)	0.6570
**FRUITS**	0.93 (0.73 - 1.20)	0.5930	0.82 (0.65 - 1.04)	0.8200	0.82 (0.65 - 1.04)	0.1070	0.92 (0.77 - 1.12)	0.4070
**NUTS**	0.84 (0.50 - 1.41)	0.5130	1.24 (0.84 - 1.84)	0.0320	1.24 (0.84 - 1.84)	0.2840	0.78 (0.54 - 1.13)	0.1850
**REFINED GRAINS**	1.16 (0.93 - 1.45)	0.1930	0.73 (0.57 - 0.94)	0.2580	0.73 (0.57 - 0.94)	0.0150	1.10 (0.94 - 1.29)	0.2200
**WHOLE GRAINS**	0.62 (0.37 - 1.07)	0.0850	0.96 (0.73 - 1.25)	0.6300	0.96 (0.73 - 1.25)	0.7430	0.94 (0.71 - 1.23)	0.6290
**SWEETS**	1.15 (0.99 - 1.33)	0.0610	0.99 (0.85 - 1.14)	0.7840	0.99 (0.85 - 1.14)	0.8770	0.97 (0.86 - 1.08)	0.5550
**JAM, MARMALADE**	0.56 (0.23 - 1.37)	0.2060	1.01 (0.59 - 1.71)	0.1690	1.01 (0.59 - 1.71)	0.9840	0.85 (0.57 - 1.28)	0.4360
**NON-ALCOHOLIC BEVERAGES**	1.12 (0.74 - 1.7)	0.5980	1.45 (1.01 - 2.08)	0.2880	1.45 (1.01 - 2.08)	0.0460	0.91 (0.63 - 1.30)	0.5890
**LIQUOR**	0.001 (0.0 - 12.7)	0.1480	0.25 (0,0 - 27.1)	0.8010	0.25 (0.0 - 27.1)	0.5600	0.14 (0.0 - 9.71)	0.3610
**COFFEE and TEA**	1.09 (0.91 - 1.30)	0.3700	0.90 (0.76 - 1.05)	0.4130	0.9 (0.76 - 1.05)	0.1840	1.03 (0.90 - 1.18)	0.6690
**PLANT OIL**	0.77 (0.60 - 0.98)	0.0350	0.85 (0.66 - 1.10)	0.8430	0.85 (0.66 - 1.10)	0.2160	1.01 (0.85 - 1.21)	0.9220
**OLIVE OIL**	1.29 (0.96 - 1.75)	0.0970	1.03 (0.78 - 1.35)	0.5300	1.03 (0.78 - 1.35)	0.8470	1.00 (0.83 - 1.21)	1.0000
**ANIMAL FAT**	1.11 (0.50 - 2.45)	0.8050	1.25 (0.73 - 2.14)	0.1310	1.25 (0.73 - 2.14)	0.4140	1.27 (0.87 - 1.86)	0.2170

Statistically significant values ($P<0.0031$) are dark grey shaded, nominally significant values ($0.01<P>0.0031$) are light grey shaded. Symptom status (with/without symptom) was used as dependent variable and food groups as independent variables in logistic regression model. We also included age, gender, physical activity, BMI and TSH levels as covariates. OR - odds ration, CI - confidence interval
